# Targeting of Deregulated Wnt/β-Catenin Signaling by PRI-724 and LGK974 Inhibitors in Germ Cell Tumor Cell Lines

**DOI:** 10.3390/ijms22084263

**Published:** 2021-04-20

**Authors:** Silvia Schmidtova, Katarina Kalavska, Veronika Liskova, Jana Plava, Svetlana Miklikova, Lucia Kucerova, Miroslava Matuskova, Lucia Rojikova, Zuzana Cierna, Adriana Rogozea, Heiko Konig, Costantine Albany, Michal Mego, Michal Chovanec

**Affiliations:** 1 Cancer Research Institute, Biomedical Research Center, Slovak Academy of Sciences, Dubravska Cesta 9, 845 05 Bratislava, Slovakia; silvia.schmidtova@savba.sk (S.S.); katarina.hainova@gmail.com (K.K.); jana.plava@savba.sk (J.P.); svetlana.miklikova@savba.sk (S.M.); lucia.kucerova@savba.sk (L.K.); miroslava.matuskova@savba.sk (M.M.); lucia.rojikova@savba.sk (L.R.); 2 Translational Research Unit, Faculty of Medicine, Comenius University, Klenova 1, 833 10 Bratislava, Slovakia; misomego@gmail.com; 3 2nd Department of Oncology, Faculty of Medicine, Comenius University and National Cancer Institute, Klenova 1, 833 10 Bratislava, Slovakia; 4 Institute of Clinical and Translational Research, Biomedical Research Center, Slovak Academy of Sciences, Dubravska Cesta 9, 845 05 Bratislava, Slovakia; veronika.liskova@savba.sk; 5 Department of Pathology, Faculty of Medicine, Comenius University, Sasinkova 4, 811 08 Bratislava, Slovakia; ciernaz@gmail.com; 6 Department of Pathology, Faculty Hospital, A. Zarnova 11, 917 75 Trnava, Slovakia; 7 Division of Hematology/Oncology, Indiana University Melvin and Bren Simon Comprehensive Cancer Center, Indiana University School of Medicine, Indianapolis, IN 46202, USA; adriana.rogozea@vwr.com (A.R.); hkonig@iupui.edu (H.K.); calbany@iu.edu (C.A.)

**Keywords:** testicular germ cell tumors, chemoresistance, Wnt/β-catenin, LGK974, PRI-724

## Abstract

The majority of patients with testicular germ cell tumors (GCTs) can be cured with cisplatin-based chemotherapy. However, for a subset of patients present with cisplatin-refractory disease, which confers a poor prognosis, the treatment options are limited. Novel therapies are therefore urgently needed to improve outcomes in this challenging patient population. It has previously been shown that Wnt/β-catenin signaling is active in GCTs suggesting that its inhibitors LGK974 and PRI-724 may show promise in the management of cisplatin-refractory GCTs. We herein investigated whether LGK-974 and PRI-724 provide a treatment effect in cisplatin-resistant GCT cell lines. Taking a genoproteomic approach and utilizing xenograft models we found the increased level of β-catenin in 2 of 4 cisplatin-resistant (CisR) cell lines (TCam-2 CisR and NCCIT CisR) and the decreased level of β-catenin and cyclin D1 in cisplatin-resistant NTERA-2 CisR cell line. While the effect of treatment with LGK974 was limited or none, the NTERA-2 CisR exhibited the increased sensitivity to PRI-724 in comparison with parental cell line. Furthermore, the pro-apoptotic effect of PRI-724 was documented in all cell lines. Our data strongly suggests that a Wnt/β-catenin signaling is altered in cisplatin-resistant GCT cell lines and the inhibition with PRI-724 is effective in NTERA-2 CisR cells. Further evaluation of Wnt/β-catenin pathway inhibition in GCTs is therefore warranted.

## 1. Introduction

Testicular germ cell tumors (GCTs) represent the most common malignancy in young males (between 20 and 34 years of age) and their incidence has steadily increased over the past few decades [1,2]. GCTs originate from primordial germ cells blocked in their differentiation, which progress towards malignant transformation via the precursor lesion called germ cell neoplasia in situ (GCNIS) [3,4]. GCTs are histologically classified into two main groups: seminomas (SE) and nonseminomas (NSE). The group of NSE consists of four different subtypes: embryonal carcinoma (EC), choriocarcinoma (ChC), yolk sac tumor and teratoma. The majority of GCTs represents tumors with more than one histology, so-called mixed germ cell tumors [5].

GCTs frequently serve as a model for a curable cancer due to their exquisite responsiveness to cisplatin-based therapy [6]. However, approximately 15–20% of all GCT patients relapse after first-line chemotherapy and require salvage treatment with cure rates of 20–60% [7,8,9,10,11,12]. Despite many efforts to improve outcomes in relapsed and chemotherapy-refractory patients, the long-term survival remains poor [13,14]. For these reasons, novel and more effective treatment options are needed to improve the clinical outcome of cisplatin-refractory patients [15].

Lines of evidence suggest that the Wnt signaling pathway plays a major role in embryogenesis as well as in the development of cancer [16]. The Wnt pathway is categorized into a canonical (β-catenin dependent) and a non-canonical (β-catenin independent) signaling pathway [17]. Persistent activation of the Wnt/β-catenin pathway has been linked to drug resistance in various cancer types [18,19]. Growing evidence suggests that the Wnt/β-catenin signaling pathway is involved in the pathogenesis and progression of GCTs and may be the contributing factor of treatment resistance [20,21,22,23,24,25]. Whole-exome and targeted sequencing on 180 cisplatin-sensitive and resistant GCTs by Bagrodia et al. reported mutations or deletions of negative regulators of the Wnt/β-catenin pathway—*AXIN1*, *APC* and *FAT1* in 8.7% of cisplatin-resistant GCTs [26]. In line with this finding, our previous study confirmed the β-catenin expression in tissue specimens from 213 out of 247 GCT patients. Intriguingly, high expression levels of β-catenin correlated with poor clinical characteristics and furthermore showed associations with an immunosuppressive microenvironment [27].

Because of the crucial role of Wnt signaling in human cancer growth and treatment resistance, Wnt-targeted treatment strategies have increasingly moved to the center of interest. To this end, multiple inhibitors targeting various components of the Wnt signaling cascade have been developed and evaluated in clinical trials [18,28,29]. For example, a phase I study of PRI-724 in patients with advanced solid tumors showed an acceptable toxicity profile of PRI-724 [30]. Results of combined treatment approach with PRI-724 and gemcitabine in a phase 1b trial concluded that this combination is safe with modest clinical activity in patients with metastatic pancreatic cancer, warranting next phase clinical trials [31,32].

PRI-724, a small molecule Wnt signaling inhibitor, was developed by PRISM Pharma (Kanagawa, Japan) to specifically target the interaction between β-catenin and its transcriptional coactivator CREB-binding protein (CBP) thereby inhibiting transcription of Wnt target genes, including survivin and cyclin D1 [33]. The Wnt-inhibiting activity of PRI-724 was evaluated in clinical trials of pancreatic cancer (NCT01764477), colorectal cancer (NCT01302405 and NCT02413853) and myeloid malignancies (NCT01606579). LGK974 is an orally administered porcupine (PORCN) inhibitor developed by Novartis (Basel, Switzerland). PORCN, the membrane bound O-acyltransferase in the endoplasmic reticulum, is essential for the secretion of Wnt ligand. LGK974 exerts its antineoplastic activity through inhibition of posttranslational acylation of WNT ligands [34]. LGK974 has been investigated in clinical trials for the treatment of solid malignancies dependent on Wnt ligands (NCT01351103, NCT02649530 and NCT02278133).

As Wnt-inhibition emerged as an innovative approach to target cisplatin-resistant GCT cells, we investigated the effects of PRI-724 and LGK972 against cell lines derived from EC, ChC and SE. We also assessed the expression of two key proteins in Wnt/β-catenin signaling pathway—β-catenin and cyclin D1 in these cell lines.

## 2. Results

### 2.1. Expression Analysis of the Wnt Signaling Pathway in Parental and Cisplatin-Resistant GCT Cell Lines

In our experiments we examined four GCT cell lines and their cisplatin-resistant variants. These resistant cells represent an in vitro model system of acquired cisplatin-resistance [35]. NTERA-2 and NCCIT are pluripotent EC cell lines, JEG-3 was derived from ChC and TCam-2 represents the only one available SE cell line. Cisplatin resistance and cross-resistance to carboplatin and oxaliplatin of cisplatin-resistant JEG-3 CisR and TCam-2 CisR cell lines were confirmed by luminescent viability assay (Figure S1, results with NTERA-2 CisR and NCCIT CisR cell lines were already published [35]). 

**Figure 1:**
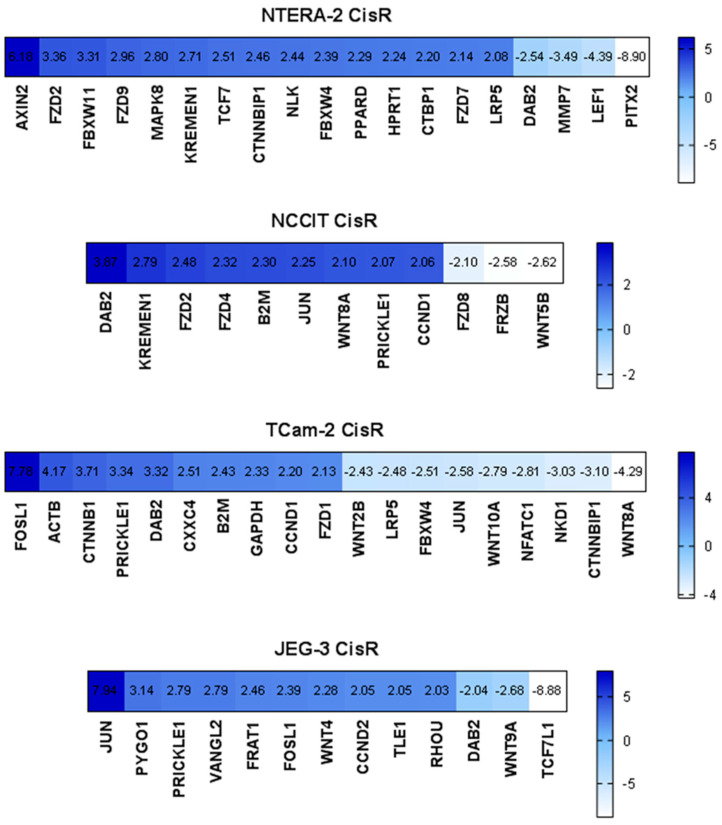
Expression analysis via RT^2^ Profiler™ PCR Array Human WNT Signaling Pathway revealed significant down- and upregulation of several genes in cisplatin-resistant GCT cell lines compared to parental cells. Visualization of fold regulation of altered genes is shown.

To determine whether cisplatin-resistance is associated with enhancement of Wnt signaling, we performed RT^2^ expression arrays, in which we analyzed the expression of Wnt signaling components (Wnt targets, ligands, receptors, β-catenin destruction complex) and genes related to Wnt signaling in parental and resistant GCT cell lines. We observed deregulation of several genes in resistant cells, including members of the “frizzled” gene family (*FZD*), *DAB2*, *JUN*, *FOSL1*, *PRICKLE1*, or Wingless-type MMTV integration site family (*WNT*). However, significant upregulation of *CTNNB1* (gene encoding β-catenin) was present only in the SE cell line TCam-2 CisR. More interestingly, we observed increased expression of *CCND1* (gene encoding cyclin D1) in the cisplatin-resistant cell lines NCCIT CisR and TCam-2 CisR and overexpression of *CCND2* (encoding cyclin D2) in JEG-3 CisR cells (Figure 1).

### 2.2. β-Catenin and Cyclin D1 Expression in Parental and Cisplatin-Resistant GCT Cell Lines

**Figure 2:**
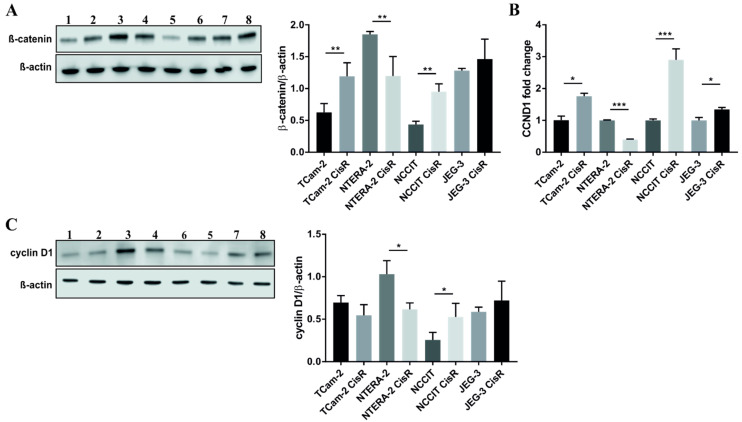
NTERA-2 CisR cells exhibited decreased levels of β-catenin and cyclin D1 compared to parental cells. (**A**) The western blot analysis of β-catenin showed decreased level of this protein in NTERA-2 CisR cells. Other GCT cell lines had increased expression of β-catenin on the protein level what was confirmed also by densitometric analysis. (**B**) Only chemoresistant NTERA-2 CisR cells exhibited the decrease in *CCND1* expression as demonstrated by qPCR. (**C**) Western blot and densitometric analysis confirmed significantly decreased expression of cyclin D1 also on the protein level. 1. TCam-2; 2. TCam-2 CisR; 3. NTERA-2; 4. NTERA-2 CisR; 5. NCCIT; 6. NCCIT CisR; 7. JEG-3; 8. JEG-3 CisR. β-actin was used as an internal loading control. * *p* < 0.05, ** *p* < 0.01, *** *p* < 0.001.

Next, we investigated protein expression levels of β-catenin and cyclin D1, two critical molecules in canonical Wnt signaling pathway. Western blot and densitometric analysis showed increased levels of β-catenin in cisplatin-resistant GCT cell lines, but not in NTERA-2 CisR cells depicted in Figure 2A and Figure S2A. qPCR analysis of *CCND1* expression confirmed the results from the RT^2^ expression array, as well as its significant decrease in NTERA-2 CisR cells (Figure 2B). A similar trend was observed in cyclin D1 protein expression levels, except for TCam-2 pair where no changes were detected. Subsequent densitometric analysis revealed a significant decrease in cyclin D1 levels in NTERA-2 CisR cells and increased levels in NCCIT CisR cells as shown in Figure 2C and Figure S2B.

### 2.3. β-Catenin Expression in GCT Xenograft Models

**Figure 3:**
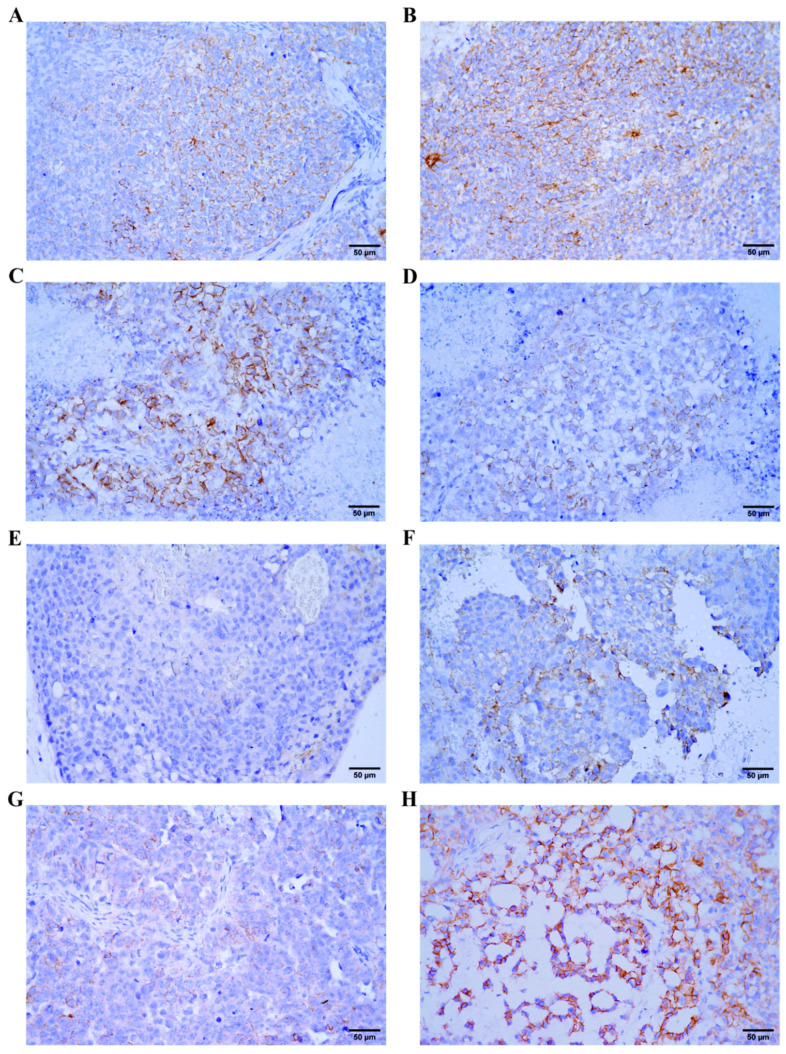
Immunohistochemical expression of β-catenin in GCT cell line xenografts. (**A**) NTERA-2, focal moderate membranous positivity (brown color) of tumor cells. (**B**) NTERA-2 CisR, strong membranous positivity (brown color) of tumor cells. (**C**) NCCIT, strong membranous positivity (brown color) of tumor cells. (**D**) NCCIT CisR, weak membranous positivity (brown color) of tumor cells. (**E**) JEG-3, negative (blue color) tumor cells. (**F**) JEG-3 CisR, moderate membranous positivity (brown color) of tumor cells. (**G**) TCam-2, weak membranous positivity (brown color) of tumor cells. (**H**) TCam-2 CisR, strong membranous positivity (brown color) of tumor cells. Original magnification ×400.

In order to explore the activity of Wnt pathway in vivo using β-catenin as a surrogate marker, we analyzed the expression of β-catenin in xenograft models using GCT cell lines. β-catenin expression was present in all GCT xenografts at different levels, representative pictures are shown in Figure 3. 

Immunohistochemical analysis of NTERA-2 and NTERA-2 CisR xenografts revealed focal moderate or strong β-catenin membranous positivity (Figure 3A,B). Similarly, strong membranous positivity was observed in NCCIT xenografts (Figure 3C). NCCIT CisR xenografts showed weak β-catenin positivity (Figure 3D). Analysis of JEG-3 xenografts revealed weak or negative β-catenin positivity (Figure 3E). Moderate membranous positivity was observed in JEG-3 CisR xenografts (Figure 3F). TCam-2 xenograft exhibited weak membranous positivity (Figure 3G) compared to TCam-2 CisR xenograft showing strong β-catenin positivity (Figure 3H).

### 2.4. Therapeutic Targeting of Wnt/β-Catenin Signaling with LGK974 and PRI-724 in Parental and Cisplatin-Resistant GCT Cell Lines

To evaluate the therapeutic potential of Wnt inhibition, we tested the antitumor effects of LGK974, a potent and specific small-molecule porcupine inhibitor, against parental and resistant cell line pairs. 

**Figure 4:**
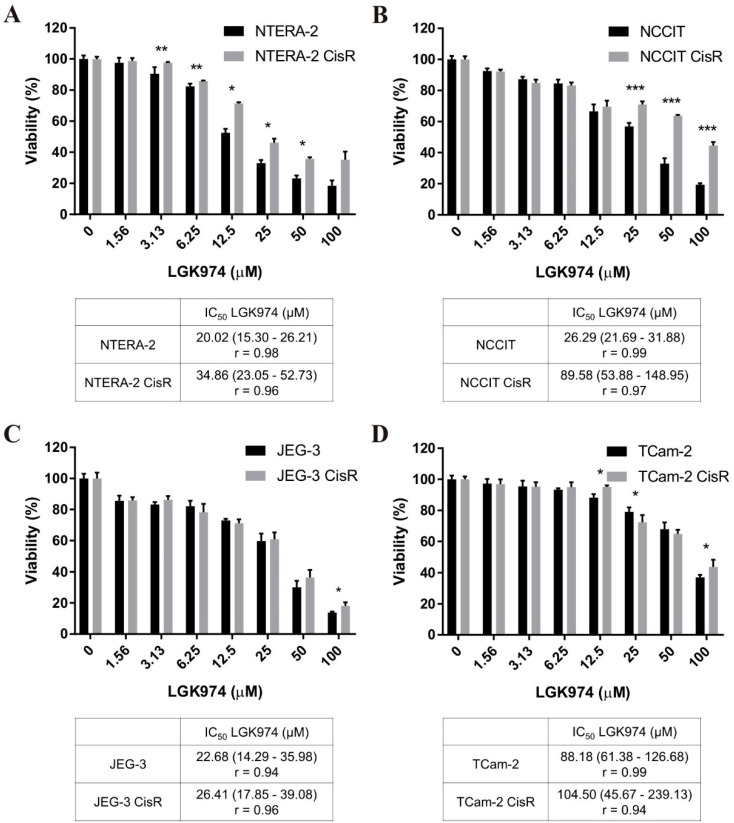
Cisplatin-resistant GCT cell lines were more resistant to LGK974 treatment compared to parental cells or there were no significant differences. (**A**–**D**) The effect of LGK974 treatment in parental and resistant GCT cell lines was determined by luminescent viability assay on day 3. Values were expressed as the averages of quadruplicates ± SD and IC_50_ values were stated in tables below graphs. * *p* < 0.05, ** *p* < 0.01, *** *p* < 0.001.

We observed dose-dependent cytotoxic effects of LGK974 in all cell lines tested. NTERA-2 CisR and NCCIT CisR cell lines were significantly more resistant to LGK974 treatment compared to parental cells (Figure 4A,B). We did not observe uniform significant changes in JEG-3 and TCam-2 pairs, and the sensitivity of parental and resistant cells to LGK974 was comparable (Figure 4C,D).

**Figure 5:**
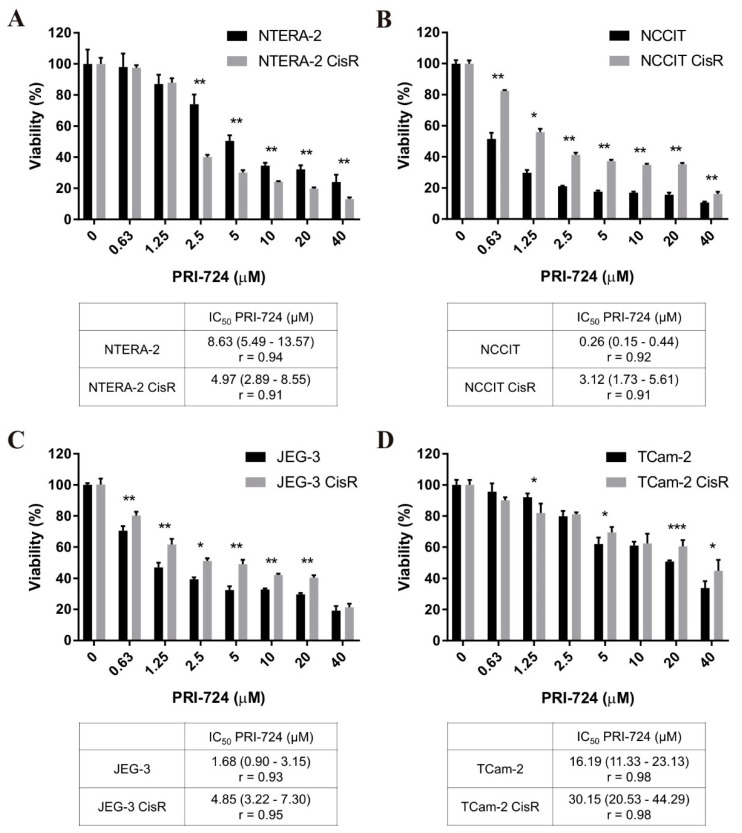
Only cisplatin-resistant NTERA-2 CisR cells were more sensitive to PRI-724 treatment compared to parental cells. (**A**–**D**) Cytotoxicity of PRI-724 in parental and resistant GCT cell lines was determined by luminescent viability assay on day 3. Values were expressed as the averages of quadruplicates ± SD and IC_50_ values were stated in tables below graphs. * *p* < 0.05, ** *p* < 0.01, *** *p* < 0.001.

Subsequently, we tested another Wnt inhibitor—PRI-724, a second generation specific CBP/catenin antagonist. Our GCT cell lines were sensitive to PRI-724 treatment in a dose-dependent manner. PRI-724 significantly decreased viability of NTERA-2 CisR cells compared to parental cells. PRI-724 at a concentration of 5 µM resulted in 50% inhibition of NTERA-2 cell viability whereas the viability of cisplatin-resistant NTERA-2 CisR cells was inhibited by 70% (Figure 5A). The IC_50_ value decreased from 8.63 µM in NTERA-2 to 4.97 µM in NTERA-2 CisR. 

The cisplatin-resistant EC cell line NCCIT CisR and ChC cell line JEG-3 CisR were significantly more resistant to PRI-724 treatment compared to parental cells (Figure 5B,C). In the case of TCam-2 pair, the parental cell line was more sensitive to PRI-724 when higher concentrations were used (Figure 5D).

### 2.5. Effect of PRI-724 Treatment on Caspase-3/7 Activity and Induction of Cell Death in Parental and Cisplatin-Resistant GCT Cell Lines

**Figure 6:**
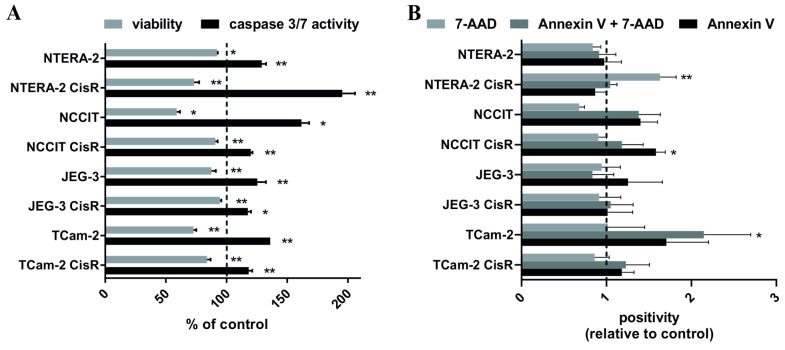
PRI-724 significantly activated caspase 3/7 and induced cell death. (**A**) Luminometric measurement of viability and caspase 3/7 activity in GCT cell lines treated with PRI-724 showed increased activity of this caspase after cisplatin PRI-724 treatment, where NTERA-2 CisR cells showed the highest activity. Values were expressed as percentage of the untreated cells (control) ± SD. (**B**) Annexin V assay revealed significant increase in population of NCCIT CisR cells in early apoptosis, increase in late apoptosis/necrosis in TCam-2 cells and necrotic population of NTERA-2 CisR cells after PRI-724 treatment. Values were expressed as relative positivity of the untreated cells (control) ± SD. 7-AAD = necrosis, Annexin V + 7-AAD = late apoptosis/necrosis, Annexin V = early apoptosis. * *p* < 0.05, ** *p* < 0.01.

In the next step, we assessed the apoptosis-inducing effects of PRI-724 in parental and resistant GCT cell lines utilizing caspase-3/7 activation assays. Activation of caspase-3/7 enzymes was observed in all treated cell lines compared to untreated controls. The highest activation was detected in NTERA-2 CisR cells, where a 27% decrease in viability resulted in 2-times higher activation of caspase-3/7 compared to untreated cells. All other cisplatin-resistant GCT cell lines exhibited an increase in caspase-3/7 activity by only ~20% (Figure 6A). Annexin V assay was performed to assess whether PRI-724 treatment increased the rate of apoptosis in parental and cisplatin-resistant GCT cell lines. We observed significantly increased number of NCCIT CisR cells undergoing early apoptosis (Annexin V positivity) after PRI-724 treatment. Late apoptosis/necrosis (Annexin V and 7-AAD double positivity) was detected in PRI-724 treated TCam-2 cells. PRI-724 treatment significantly increased population of necrotic (7-AAD positive) NTERA-2 CisR cells (Figure 6B).

### 2.6. Effect of PRI-724 Treatment on Migration of Cisplatin-Resistant GCT Cell Lines

**Figure 7:**
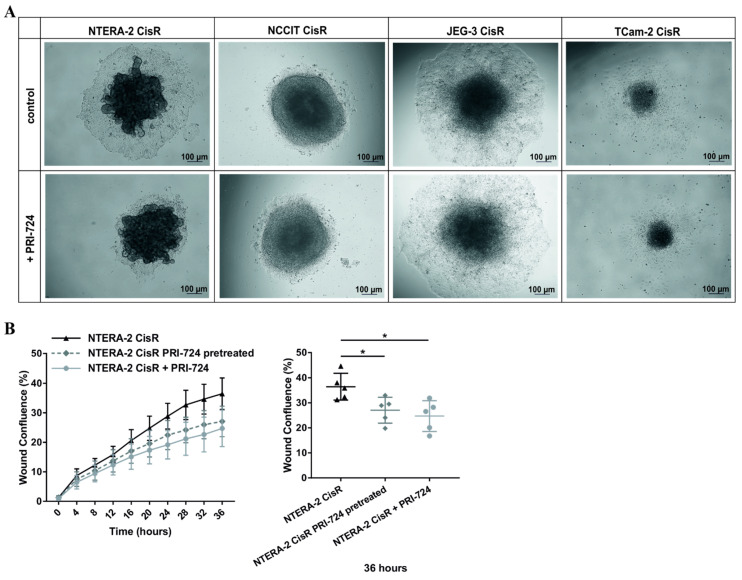
PRI-724 significantly inhibited migratory capacity of NTERA-2 CisR cells. (**A**) PRI-724 significantly decreased the migration of NTERA-2 CisR cells out of the 3D spheroid. Migratory capacity of other GCT cell lines was not affected by PRI-724 treatment. Images were taken 3 days post transfer from non-adherent to adherent conditions. (**B**) Migration of NTERA-2 CisR cells pretreated (for 72 h before plating) or treated (for 36 h) with PRI-724 was significantly decreased compared to untreated NTERA-2 CisR cells in a wound healing assay. Confluent monolayers of NTERA-2 CisR cells were wounded and cell migration was observed by live-cell imaging for 36 h. * *p* < 0.05.

To investigate whether PRI-724 inhibitor could reduce the migratory capacity of cisplatin-resistant GCT cell lines, we performed 3D migration assays. All GCT cell lines—parental and resistant—were able to form 3D multicellular spheroids (Figure S3, results with NTERA-2 CisR and NCCIT CisR cells were already published [35]). Unlike in other cisplatin-resistant GCT cell lines, PRI-724 treatment negatively affected migration of NTERA-2 CisR cells from 3D spheroid as depicted in Figure 7A. To confirm the inhibitory effect of PRI-724 on migratory capacity of NTERA-2 CisR cells, we performed wound healing assay. For this experiment we used two types of PRI-724 treatment: (1) NTERA-2 CisR cells pretreated with PRI-724 for 72 h, (2) NTERA-2 CisR cells treated with PRI-724 after the wound was scratched. In both cases we observed significantly decreased migration compared to untreated NTERA-2 CisR cells (Figure 7B and Figure S4).

### 2.7. Effects of Combined Treatment with PRI-724 and Cisplatin in NTERA-2 CisR Cells

**Figure 8:**
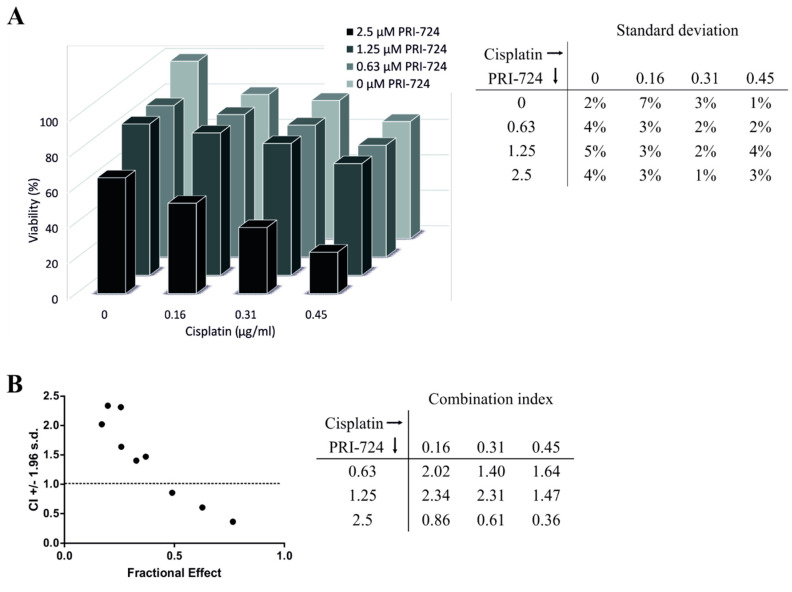
Cisplatin decreased viability of NTERA-2 CisR spheroids in combination with high concentration of PRI-724. (**A**) The effect of combined treatment with PRI-724 and cisplatin in NTERA-2 CisR multicellular spheroids. Relative viability was determined by luminescent viability assay on day 6. Values were expressed as the averages of hexaplicates and SD were indicated in the table. (**B**) Data obtained by luminometric assay were analyzed by Calcusyn software and Fa-CI plot was created. Plot displays synergism (CI < 1), additivity (CI = 1) or antagonism (CI > 1) for the entire spectrum of effects [36]. CI values were indicated in the table. CI—function of effect level, Fa—fraction affected (Fa = 1 − % of viable cells/100).

Since the data from our viability assays indicated that PRI-724 is more effective than LGK974, we focused on PRI-724 to assess the activity of combined treatment approaches. To test whether PRI-724 and cisplatin yields synergistic effects, we treated NTERA-2 CisR multicellular spheroids with this combination. We observed synergistic effects only when higher concentrations of PRI-724 were used. The viability of the NTERA-2 CisR spheroids decreased by 35% upon the treatment with 0.45 μg/mL cisplatin alone. However, 2.5 μM PRI-724 with 0.45 μg/mL cisplatin achieved a 77% reduction in tumor cell viability (Figure 8A). The combination index (CI) was above 1 indicating more antagonistic effect of PRI-724 and cisplatin when lower PRI-724 concentrations were used. Only 2.5 μM PRI-724 in combination with cisplatin led to synergistic effects (Figure 8B).

### 2.8. β-Catenin and Cyclin D1 Expression in PRI-724 Treated Cisplatin-Resistant GCT Cell Lines

**Figure 9:**
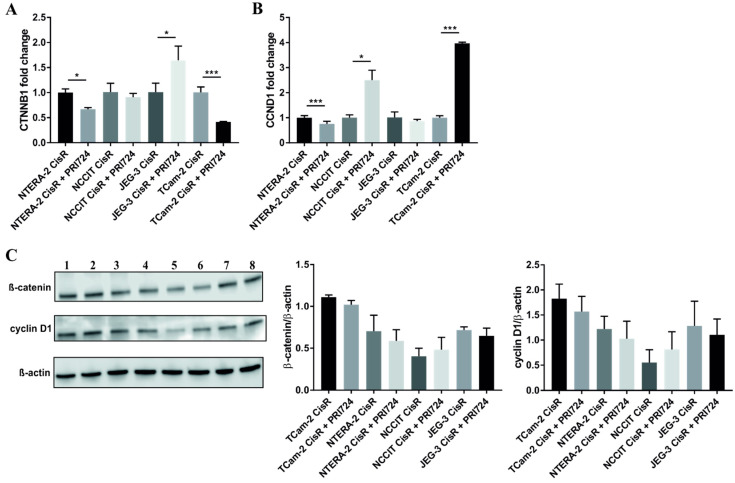
PRI-724 treatment did not affect protein levels of β-catenin and cyclin D1 in GCT cell lines. (**A**) qPCR analysis revealed significant decrease in expression of *CTNNB1* in NTERA-2 CisR and TCam-2 CisR cells and increased *CTNNB1* expression in JEG-3 CisR cells treated with PRI-724 inhibitor. (**B**) Decreased *CCND1* expression was confirmed in NTERA-2 CisR cells after PRI-724 treatment. Significant upregulation was detected in PRI-724 treated NCCIT CisR and TCam-2 CisR cells. (**C**) Western blot and densitometric analysis confirmed that PRI-724 treatment did not change the protein levels of β-catenin and cyclin D1 in GCT cell lines. 1. TCam-2 CisR; 2. TCam-2 CisR + PRI-724; 3. NTERA-2 CisR; 4. NTERA-2 CisR + PRI-724; 5. NCCIT CisR; 6. NCCIT CisR + PRI-724; 7. JEG-3 CisR; 8. JEG-3 CisR + PRI-724. β-actin was used as an internal loading control. * *p* < 0.05, *** *p* < 0.001.

Next, we examined how PRI-724 treatment affects the expression of our two key proteins, β-catenin and cyclin D1, in cisplatin-resistant GCT cell lines. Analysis of gene expression via qPCR revealed significant decrease in *CTNNB1* expression in NTERA-2 CisR and TCam-2 CisR cells after PRI-724 treatment. We observed *CTNNB1* overexpression in PRI-724 treated JEG-3 CisR cells (Figure 8A). Expression of *CCND1* was also significantly decreased in NTERA-2 CisR cells after PRI-724 treatment. Increased expression of *CCND1* was detected in NCCIT CisR and TCam-2 CisR cells treated with PRI-724 (Figure 9B). However, results obtained by western blotting showed that the inhibition of CBP/β-catenin complex by PRI-724 did not affect overall protein levels of β-catenin and its downstream target cyclin D1 (Figure 9C and Figure S5).

### 2.9. In Vivo Efficacy of PRI-724 in NTERA-2 CisR Xenograft Model

**Figure 10:**
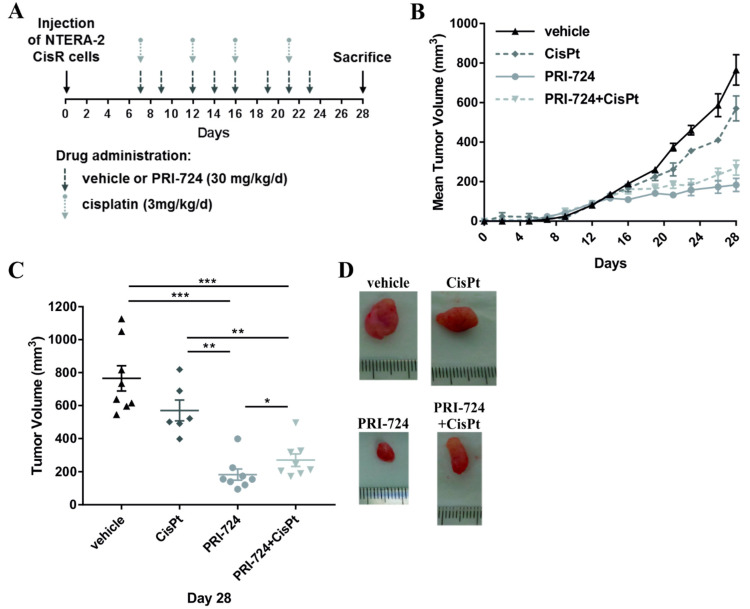
PRI-724 exhibited antitumorigenic effect on NTERA-2 CisR xenografts in vivo. (**A**) Outline scheme of the treatment. Cisplatin, PRI-724 and vehicle were intraperitoneally administered to mice. The timing of drug administration is indicated by arrows. (**B**) PRI-724 significantly inhibited the growth of NTERA-2 CisR xenografts. (**C**) The tumor sizes were significantly smaller in the group of mice treated with PRI-724 alone compared to control group (vehicle) or to mice treated with cisplatin. Cisplatin did not enhance antitumorigenic effect of PRI-724. (**D**) Images of representative tumors at the end of the experiment showing the antitumorigenic effect of Wnt/β-catenin/CBP inhibition by PRI-724. * *p* < 0.05, ** *p* < 0.01, *** *p* < 0.001.

In order to determine the effects of Wnt inhibition by PRI-724 on tumor growth in vivo, xenograft models were generated utilizing the NTERA-2 CisR cell line which was the most sensitive to PRI-724 treatment in our experiments in vitro. Cells were injected s.c. into mice flanks to produce tumor xenografts and animals were divided into 4 treatment groups: (1) untreated control/vehicle (n = 4), (2) cisplatin—3 mg/kg/d (n = 3), (3) PRI-724—30 mg/kg/d (n = 4), (4) and combined therapy with PRI-724 and cisplatin (n = 4). All mice developed palpable tumors between the 5th and 7th day after the inoculation, and treatment commenced on day 7 (Figure 10A). Multivariate analysis of repeated measures showed that cisplatin treatment did not significantly affect tumor growth. In contrast, PRI-724 alone significantly inhibited growth of NTERA-2 CisR xenografts (the mean of tumor volume was 183 mm^3^) when compared to the control group (765 mm^3^). However, cisplatin treatment did not augment this inhibition (Figure 10B,C) which was in line with our in vitro data showing an antagonistic effect of this combination. Representative pictures of xenografts are shown in Figure 10D.

## 3. Discussion

GCTs represent a remarkable example of cancer. Due to the discovery of exceptional sensitivity to cisplatin-based treatment, once fatal metastatic GCTs became a curable disease. Cisplatin-based combination chemotherapy increased the 5-year survival from less than 10% to more than 80% in patients with advanced disease over the last four decades [37].

However, for patients with cisplatin-resistant disease (approximately 30% of patients [38]) the curative treatment options are lacking [39]. Targeted approaches including treatment with everolimus [40,41], bevacizumab [42], sunitinib [43] or immune-check point inhibitors [44,45] failed or showed limited effects in patients with chemoresistant disease. An improved understanding of the mechanisms involved in the development of cisplatin resistance in GCTs holds the key for the development of more effective treatment approaches. Previously, we reviewed available cell line models suitable for the evaluation of therapeutic strategies in vitro and to study molecular mechanisms involved in hypersensitivity and chemoresistance of GCTs [46,47]. Since then, we generated more cisplatin-resistant variants derived from GCT cell lines. To examine the effect of Wnt/β-catenin inhibitors NTERA-2 and NCCIT pairs were used as representatives for EC, JEG-3 for ChC and TCam-2 cells for SE. Testicular teratoma and yolk sac tumor cell lines were unavailable.

To analyze the effect of Wnt inhibition on cell viability in our GCT cell lines, we treated cells with the PORCN inhibitor LGK974. This inhibitor was previously tested in both in vitro and in vivo preclinical cancer models and showed effective inhibition of cancer cell growth, migration, invasivity or formation of metastasis. For example, treatment of neuroendocrine tumor cell lines with LGK974 significantly decreased cell viability [48]. LGK974 inhibited proliferation and colony formation and induced apoptosis in clear cell renal cell carcinoma cells. Moreover, LGK974 treatment also suppressed their migration and invasion [49]. In addition, synergistic effect of this inhibitor with chemotherapeutic drugs was shown in glioblastoma [50] and ovarian cancer [51]. In vivo studies confirmed that LGK974 can effectively inhibit xenograft tumor growth and reduce the metastatic spread of cancer cells [34,52,53].

Parental and resistant GCT cell lines used in our study were sensitive to LGK974 treatment in a dose-dependent manner. However, none of the resistant cell lines was more sensitive to this treatment compared to parental cells. Published data suggest that upstream Wnt pathway inhibitors such as PORCN inhibitors are less effective in cancer cells with mutations of downstream Wnt pathway components such as APC mutations [18]. The presence of APC mutations was confirmed in 8.7% of platinum-resistant GCTs [26]. We hypothesized that mutations of APC could at least in part account for the limited response of GCT cells to LGK974 treatment, but our current knowledge is insufficient and further research is warranted.

Preclinical studies showed that PRI-724, the second-generation of CBP/catenin antagonist, inhibited cell proliferation and reduced cell growth in a variety of cancer types, including neuroendocrine tumors [48], osteosarcoma [54], head and neck carcinoma [55], hepatocellular carcinoma [56] and also in soft tissue sarcomas [57]. Similarly to LGK974, we showed that GCT cell lines were sensitive to PRI-724 in a dose-dependent manner. In our study, however, only the cisplatin-resistant EC cell line NTERA-2 CisR displayed increased sensitivity to treatment compared to parental NTERA-2 cells. JEG-3 CisR and TCam-2 CisR cells, and the EC NCCIT CisR cell line, were more resistant than parental cells. Our observations could at least in part be explained by different developmental origin of NTERA-2 and NCCIT cell lines. NTERA-2 cell line was originally isolated from the lung metastasis which was obtained from a 22-year old patient with primary testicular EC. The tumor was then xenografted onto mouse as TERA-2 cell line. TERA-2 tumor cells were subsequently cloned into the NTERA-2 cell line retaining the capacity to differentiate into diverse solid tissues. [58]. The NTERA-2 cell line represents a model of pluripotent embryonal cancer stem cell derived from testicular EC. In clinical practice, testicular EC remains a model for cancer cure with excellent cure rates [59]. We presume the NTERA-2 to resemble this good prognosis testicular EC variant. On the other hand, NCCIT cell line represents a pluripotent human testicular embryonal carcinoma derived from a primary mediastinal nonseminomatous GCT (PMNSGCT) [60,61]. PMNSGCTs are rare, clinically aggressive, and carry an inferior prognosis compared to primary gonadal NSE [62]. Long-term cure is achieved in about 40% of all patients with PMNSGCTs treated in expert academic centers [63], while updated IGCCCG (The International Germ Cell Cancer Collaborative Group) analysis has shown 67%, 89% and 96% 5-year overall survival in poor, intermediate and good-risk nonseminomas [64]. Considering the clinical course of disease, there is a significant disconnect between testicular nonseminoma and PMNSGCT. Hence, we hypothesize the variant underlying molecular background in EC derived from testis and anterior mediastinum, and, consequently the resulting different treatment efficacy of PRI-724 in NTERA-2 versus NCCIT cell lines. Treatment with LGK-974 provided similar results in NTERA-2 and NCCIT cell lines producing some effect in parental cell lines but showing increased resistance in CisR variants. While we consider NTERA-2 and NCCIT different in terms of molecular landscape and clinical behavior of their counterparts in the real-life clinical setting, they still represent an EC cell with common basic characteristics. In our opinion, certain differences between NTERA-2 and NCCIT do not preclude some treatments to have similar effect, while others may be less effective in one over the other. Their biological differences should be further researched for better understanding.

Due to the significant efficacy of PRI-724 treatment in NTERA-2 CisR cells, we tried to further sensitize these cells using a combined treatment approach with cisplatin. Previous studies of combined treatments with PRI-724 showed its ability to enhance the cytotoxic effect of chemotherapeutic drugs in platinum-resistant ovarian cancer cells [65] as well as in soft tissue sarcomas [57]. Our in vitro data showed synergistic effect only when higher PRI-724 concentrations were used. However, this synergy was not confirmed in in vivo experiments. Nevertheless, this CBP/catenin antagonist alone showed significant anti-tumorigenic effect and decreased the growth of NTERA-2 CisR xenografts. To our knowledge, the combination of PRI-724 and cisplatin was not yet explored in clinical studies. Based on the results of our in vitro and in vivo study, we hypothesize that PRI-724 monotherapy could provide meaningful efficacy while being less toxic than combination with cisplatin in humans. A phase I dose escalation study assessed safety and tolerability of PRI-724 monotherapy in 14 patients with Hepatitis-C induced liver cirrhosis. PRI-724 was given in doses up to 160 mg/m^2^ in a continuous infusion 1 week on and 1 week off for 6 cycles. The most common side effects were nausea (29%) and fatigue (21%). The lower dosing regimens of 10 and 40 mg/m^2^ were tolerated well [66]. The safety of PRI-724 assessed in a study of 18 patients with various cancers using escalating dosing regimen from 640 mg/m^2^ 1 week on and 1 week off. The recommended phase 2 dose was 905 mg/m^2^. There was one dose limiting toxicity (DLT) of grade 3 hyperbilirubinemia, 2 patients had non-DLT grade 3 hyperbilirubinemia. Grade 2 adverse events were diarrhea (11%), hyperbilirubinemia (11%), hypophosphatemia (11%), nausea (6%), fatigue (6%), anorexia (6%), thrombocytopenia (6%) and alkaline phosphatase elevation (6%) [30]. Treatment with PRI-724 monotherapy therefore seems to be well tolerated and a phase II study in cancer patients is warranted.

The activity of PRI-724 was also associated with an increased expression of apoptosis-related proteins and the induction of apoptosis in hepatocellular and head and neck carcinoma cells [55,56]. We observed an increased activity of caspase-3/7 in parental and resistant GCT cell lines treated with PRI-724. The highest activity of this enzyme was observed in the NTERA-2 CisR cell line, which was significantly more sensitive to treatment when compared to parental cells. Nevertheless, analysis of cell death using Annexin V assay showed significant induction of apoptosis only in TCam-2 and NCCIT CisR cells treated with PRI-724. Annexin V positivity correlating with induction of apoptosis was detected in NCCIT CisR cell line. We observed late apoptosis/necrosis (7-AAD and Annexin V double positivity) in TCam-2 cell line 72 h after PRI-724 treatment. Significant increase in 7-AAD positivity revealed necrotic cell death in NTERA-2 CisR cells treated with PRI-724 inhibitor. Appropriate timing of apoptosis assays is extremely important [67]. It could be possible that 72 h of PRI-724 treatment is not suitable to correctly detect apoptosis using these types of assays and further experiments are warranted.

We further found a positive correlation between treatment sensitivity and migration. PRI-724 negatively affected cell migration from 3D spheroids to a certain extent in NTERA-2 CisR cells while the migration of other cisplatin-resistant GCT cell lines was not decreased. Moreover, we confirmed negative effect of PRI-724 on cell migration of NTERA-2 CisR cells also in scratch wound assay. Significant inhibitory effects on cell migration, invasion, or colony formation were found in different cancer cell line models [54,55].

Previous studies suggested that Wnt/β-catenin signaling may be involved in cisplatin resistance of many cancer types, including GCTs [26]. Increased expression of β-catenin was observed in cisplatin-resistant cell lines, including lung adenocarcinoma [68], oral squamous cell carcinoma [69] and ovarian cancer [65,70]. In addition, elevated β-catenin activity contributed to carboplatin resistance in ovarian cancer cells [71]. The role of β-catenin in cisplatin resistance, relapse and prognosis was confirmed in head and neck squamous cell carcinoma [72]. Moreover, we have previously shown a significant association between increased β-catenin expression in GCTs and poor clinical characteristics, including intermediate/poor risk disease and high serum tumor marker expression [27]. Another work has shown that high β-catenin expression in primary testicular GCTs was associated with relapse and NS-GCT histology in clinical stage I disease. This finding may help refining the approach to risk stratification of stage I GCTs [73]. Western blot analysis in our GCT cell lines revealed significantly increased levels of β-catenin protein expression in two cisplatin-resistant cell lines with the exception of NTERA-2 CisR cells where the expression was significantly decreased. We also confirmed β-catenin expression in xenograft models using GCT cell lines via immunohistochemical staining.

Wang et al. have shown that increased expression of β-catenin but also cyclin D1 overexpression correlated with poor overall survival in ovarian serous carcinomas [74]. Significantly lower progression-free and overall survival were observed in cyclin D1-positive multiple myeloma patients [75]. Upregulation of *CCND1* and *CCND3* genes was associated with cisplatin resistance in human oral squamous cell carcinoma cell lines [76]. The expression analysis of three pairs of parental and cisplatin-resistant GCT cell lines revealed that *CCND1* was the most significantly differentially expressed gene. Further analysis of clinical samples identified *CCND1* overexpression in the majority of cisplatin-resistant GCTs as well as its involvement in cisplatin resistance of ovarian and prostate cancer [77]. We observed increased expression of cyclin D1 on mRNA or protein level in NCCIT CisR, JEG-3 CisR and TCam-2 CisR cells. Again, NTERA-2 CisR cell line was the only exception and significant *CCND1* downregulation was confirmed by qPCR and Western blotting assays. These findings are consistent with observations within the three pairs of parental and resistant GCT cell lines, where cisplatin-resistant EC cell line 833KR had decreased expression of this gene [77].

Furthermore, Wnt signaling pathway has been previously suggested as an intrinsic mechanism of inhibiting the T-cell infiltration in tumors. Our previous work has demonstrated an association between β-catenin expression and suppressed immune environment. High PD-L1 expression on tumor cells was predictive of poor outcome in GCTs [78]. Moreover, we have shown a significant correlation between the expression of PD-L1 on tumor cells and β-catenin expression. Patients with low β-catenin had lower PD-L1 expression on tumor cells compared to patients with high β-catenin. Increased β-catenin expression also correlated with low systemic immune-inflammation index [27]. Chen et al. performed a comprehensive profiling to analyze the correlation between *CCND1* amplification and the prognosis and the response to immune checkpoint inhibitors (ICIs). *CCND1* amplification was associated with a decreased overall survival in a cohort of melanoma patients as well as in patients with solid tumors. It was also related to immunosuppression in the tumor microenvironment [79]. Poor response to toripalimab, a humanized IgG4 monoclonal antibody (mAb) against PD-1, correlated with *CCND1* amplification in melanomas [80]. Based on whole-exome sequencing and RNA-sequencing profiling, *CCND1* gain was detected in patients with melanoma resistant to anti-PD-1 immunotherapy [81]. Xiong et al. analyzed genomics, transcriptomics, and immunogenicity of two patients with hyperprogressive disease (HPD) with accelerated tumor growth after anti-PD-1 immunotherapy. Ingenuity Pathway Analysis identified activation of *CCND1*, *MYC*, and *VEGF* oncogenes in these two samples [82]. Amplification of several genes located on chromosome 11q13, including *CCND1*, was detected in 5 patients with different solid tumors experiencing HPD [83]. Altogether, these data highly suggest an association of Wnt/β-catenin/cyclin D1 signaling with the immune suppressive microenvironment and resistance to ICIs. The development of pre-clinical GCT models to explore the immune-related associations, however, is extremely challenging.

Decreased expression of the target genes including *CCND1* after treatment with PRI-724 or LGK974 was previously observed. PRI-724 treatment led to decreased protein levels of the Wnt target cyclin D1 in human osteosarcoma cells [54]. Downregulation of *CCND1* and *CDC25A* genes was also observed in soft tissue sarcoma cell lines [57]. After treatment of clear cell renal cell carcinoma cells with LGK974, the expression levels of β-catenin, cyclin D1, c-Myc, *MMP9*, and *MMP2* were significantly decreased [49]. Expression of c-Myc and cyclin D1 was also downregulated in neuroendocrine tumor cell lines treated with LGK974. In our experiments, Wnt/β-catenin inhibition via PRI-724 treatment decreased expression of *CTNNB1* and *CCND1* in NTERA-2 CisR cells. *CTNNB1* expression was downregulated also in TCam-2 CisR cells, but expression of *CCND1* was increased. We observed increased *CTNNB1* expression in JEG-3 CisR cells after PRI-724 treatment and also overexpression of *CCND1* in NCCIT CisR cells. However, PRI-724 did not affect protein levels of β-catenin or cyclin D1.

Our findings suggest that Wnt/β-catenin/cyclin D1 signaling cascade could be involved in disease progression and/or cisplatin resistance of GCTs. A major limitation of our study is the limited number of cell lines representing each GCT histological subtype. Another limitation is a small number of tissue samples used for immunohistochemistry staining in each individual GCT subtype. Therefore, the statistical analysis of the differences in β-catenin expression between all parental and cisplatin-resistant tumors and the correlation of β-catenin expression in tissue with matching GCT cell line clones treated in vitro was not possible. The effect of Wnt/β-catenin inhibition via PRI-724 treatment was the most prominent in our cisplatin-resistant NTERA-2 CisR cell line. NTERA-2 was the only cell line showing down-regulated *CTNNB1* and *CCND1* genes in the CisR variant. Despite this phenomenon, we have still seen the increased treatment effect in NTERA-2 CisR cells. This may be explained by different effects of *CCND1* down-regulation in cancer cells. While up-regulation of *CCND1* is commonly associated with increased migratory capacity, chemotherapy resistance and poor outcomes in various cancers, the down-regulation of *CCND1* in breast cancer has been linked to excessively infiltrative growth and poor prognosis [84]. This suggests that *CCND1*-specific effect is very complex and not mediated by single pathway, which, perhaps, is the reason for difference in NTERA-2 WNT/β-catenin signaling compared to other GCT cell lines. PRI-724 also down-regulated the expression of *CTNNB1* and *CCND1* on mRNA level, which confirms the on-target activity of this inhibitor. However, protein levels of β-catenin and cyclin D1 after PRI-724 treatment were not changed.

There is also a significantly different effect of PRI-724 treatment in cisplatin-resistant EC cell lines NTERA-2 CisR and NCCIT CisR that could be explained by the different origin of these cells. We have previously shown that these cell lines exhibit increased expression of cancer stem cell (CSC) markers including overexpression of different aldehyde dehydrogenase (ALDH) isoforms and overall ALDH activity [35]. Specific CBP/β-catenin antagonists, including PRI724, appear to have the ability to safely eliminate CSCs [85]. Significantly higher ALDH activity was observed in NTERA-2 CisR cells compared NCCIT CisR cell line [35]. These results potentially suggest greater enrichment for CSCs in NTERA-2 CisR cells and the possible explanation of PRI-724 cytotoxicity. Another explanation could be that the Wnt/β-catenin signaling may represent an independent or partially independent mechanism for progression and treatment resistance of GCTs, therefore the NTERA-2 CisR and NCCIT CisR cell lines should be further studied as separate models of chemoresistant GCTs. Further experiments will be needed to fully understand the intricacies of Wnt/β-catenin pathway in GCTs.

## 4. Materials and Methods

Chemicals were purchased from Sigma-Aldrich (Saint-Louis, MO, USA) if not stated otherwise.

### 4.1. Cell Cultivation

The human embryonal carcinoma cell line NTERA-2 (ATCC^®^ CRL-1973™) and choriocarcinoma cell line JEG-3 (ATCC^®^ HTB-36™) were maintained in high-glucose (4.5 g/L) DMEM (PAA Laboratories GmbH, Pasching, Austria) containing 10% FBS (GIBCO^®^ Invitrogen, Carlsbad, CA, USA), 10,000 IU/mL penicillin (Biotica, Part. Lupca, Slovakia), 5 μg/mL streptomycin, 2.5 μg/mL amphotericin and 2 mM glutamine (PAA Laboratories GmbH).

The human testicular seminoma cell line TCam-2 (kindly provided by Kitazawa, Ehime University Hospital, Shitsukawa, Japan) and embryonal carcinoma cell line NCCIT (ATCC^®^ CRL-2073™) were cultivated in RPMI (GIBCO^®^ Invitrogen, Carlsbad, CA, USA) containing 10% FBS, 10,000 IU/mL penicillin, 5 μg/mL streptomycin, 2.5 μg/mL amphotericin and 2 mM glutamine. Cells were cultivated at 37 °C in humidified atmosphere and 5% CO_2_.

Cisplatin-resistant variants of parental cell lines, designated as CisR, were all derived by propagating the cells in increasing concentrations of cisplatin (Hospira UK Ltd., Warwickshire, UK) for 6 months as described previously [35,86]. Briefly, exponentially growing cells were exposed to 0.05 µg/mL cisplatin initially. When the cells started to expand, the concentrations were gradually increased to 0.1 µg/mL, respectively in case of JEG-3 cells to 0.2 µg/mL.

### 4.2. Viability Assays

Quadruplicates of cells were plated at 3 × 10^3^–5 × 10^3^ cells/100 μL media per well and were seeded in 96-well white-walled plates (Corning Costar Life Sciences, Amsterdam, The Netherlands) overnight. For the evaluation of sensitivity to tested inhibitors, cells were seeded in 96-well plates overnight and treated with PRI-724 (SelleckChem, Houston, TX, USA; 0.31–40 μM) or LGK974 (SelleckChem, 1.56–100 μM). Stock solutions were prepared by dissolving in DMSO and stored at −80 °C according to manufacturer instructions. Relative viability of the cells was determined by the CellTiter-Glo™ Luminescent Cell Viability Assay (Promega Corporation, Madison, WI, USA) and evaluated by the GloMax Discover System reader (Promega Corporation) after 3 days of treatment. Experiments were performed in quadruplicates at least three times with similar results and the representative result is shown. Values were expressed as means ± SD and IC_50_ values were calculated by CalcuSyn 1.1 software (Biosoft, Cambridge, UK).

The efficacy of combined treatment with cisplatin (0.16–0.45 μg/mL) and PRI-724 (0.63–2.5 μM) in NTERA-2 CisR 3D multicellular spheroids was evaluated by the CellTiter-Glo™ 3D Cell Viability Assay (Promega Corporation). 3D multicellular spheroids were prepared in hexaplicates of NTERA-2 CisR (5 × 10^3^ cells/well) and seeded into 96-well ultra-low attachment plates (Corning 7007, Corning Inc., Corning, NY, USA) in 100 µL of culture medium (as described above). Medium containing both drugs was added at the same time three days after plating in 96-well ultra-low attachment plates. Cells were cultured for next 6 days and combinational effect of drugs was calculated according to Chou [36]. Briefly, combination index (CI) was computed for every affected fraction (fa, proportion of dead cells): CI < 1 represents synergism, CI = 1 additivity and CI > 1 antagonism. Calcusyn software was used for analysis [87].

### 4.3. Caspase Assay

Quadruplicates of cells were plated at 3 × 10^3^–5 × 10^3^ cells/100 μL media per well and were seeded in 96-well white-walled plates overnight. PRI-724 diluted in culture media (NTERA-2 pair: 1.25 μM; NCCIT and JEG-3 pairs: 0.63 μM; TCam-2 pair: 2.5 μM) was added to the cells for 3 days and caspase-3/7 activity was determined by the Caspase-Glo^®^ 3/7 Assay (Promega Corporation) on GloMax Discover System (Promega Corporation). Same procedure of cultivation was used to determination of cell viability by the CellTiter-Glo™ Luminescent Cell Viability Assay as described above. Values were determined as mean values of % of control ± SD, where control represented viability or caspase-3/7 activity of untreated cells.

### 4.4. Annexin V Assay

Cells were seeded on 24-well plates (3 × 10^4^/well) overnight and treated with PRI-724 for 72 h (NTERA-2 pair: 1.25 μM; NCCIT and JEG-3 pairs: 0.63 μM; TCam-2 pair: 2.5 μM). Harvested cells (also the ones from supernatant) were washed in PBS. Cell pellets were subsequently resuspended in Binding Buffer containing PE-conjugated Annexin V (eBioscience, San Diego, CA, USA) and incubated for 15 min at room temperature, protected from light. 7-AAD (2 μg/mL, Sigma Aldrich) was added to stain non-viable cells. Analysis was performed on BD FACSCanto™ II flow cytometer (Becton Dickinson, Franklin Lakes, NJ, USA), data were analyzed with FCS Express program (De Novo Software, Glendale, CA, USA).

### 4.5. 3D Migration Assay

3D multicellular spheroids were prepared in octaplicates of 5 × 10^3^ NTERA-2 and TCam-2 parental and resistant cells, or 3 × 10^3^ NCCIT and JEG-3 parental and resistant cells. Three days after plating in 96-well ultra-low attachment plates, spheroids were placed on top of a conventional cell culture 96-well plate (CytoOne, USA Scientific, Inc., Ocala, FL, USA) into culture medium or medium containing PRI-724 inhibitor (NTERA-2 pair: 1.25 μM; NCCIT and JEG-3 pairs: 0.63 μM; TCam-2 pair: 2.5 μM). After attachment of the spheroid to the plastic surface cells started to migrate. Digital images were captured and analyzed with Axiovert 40C Zeiss microscope using the Zen 2.6 software (Carl Zeiss Microscopy GmbH, Jena, Germany) 3 days after placing to the plate.

### 4.6. Wound Healing Assay

Thirty thousand of NTERA-2 CisR cells (untreated or pretreated with PRI-724 inhibitor for 72 h) per well were plated in pentaplicates in ECM-coated ImageLock 96-well plates (Essen BioScience, Royston, UK) and let to adhere overnight. Confluent monolayers were wounded with wound making tool (Essen BioScience), washed and supplemented with serum-free culture medium with or without PRI-724 inhibitor (1.25 μM). Cell migration was evaluated by IncuCyte^®^ Scratch Wound Cell Migration and Invasion System and documented by the IncuCyte ZOOM™ kinetic imaging system (Essen BioScience). Results were based on the relative wound density measurements and expressed as means of three independent experiments run in pentaplicates ± SD.

### 4.7. Gene Expression Array

RT^2^ Profiler™ PCR Array Human WNT Signaling Pathway (PAHS-043Z, Qiagen, Hilden, Germany) was used for analysis of the expression of Wnt signaling components and genes related to Wnt signaling in parental and resistant cell lines. Cell pellets were prepared from cultured cells collected by trypsinization and RNA was isolated by AllPrep RNA/Protein kit (Qiagen). RNA was then reverse-transcribed with RT^2^ First Strand Kit (Qiagen). Arrays were performed using RT^2^ SYBR Green Mastermix (Qiagen) according to manufacturer’s instructions on CFX96™ Touch Real-Time PCR Detection System (BIO-RAD Laboratories, Hercules, CA, USA). Results were obtained via web portal http://www.qiagen.com/geneglobe (accessed date–25 January 2021).

### 4.8. qPCR Expression Analysis

Cultured cells (untreated or treated with PRI-724 for 3 days (NTERA-2 pair: 1.25 μM; NCCIT and JEG-3 pairs: 0.63 μM; TCam-2 pair: 2.5 μM)) were collected by trypsinization and total RNA was isolated by NucleoSpin^®^ RNA II (Macherey-Nagel, Düren, Germany) and treated with RNase-free DNase (Qiagen). Total RNA was subjected to control PCR to confirm the absence of genomic DNA contamination. RNA was reverse transcribed with RevertAid™ H minus First Strand cDNA Synthesis Kit (Thermo Fisher Scientific Inc., Waltham, MA, USA). Following protocol was used: activation step at 95 °C for 3 min, 40 cycles of denaturation at 95 °C for 45 s, 30 s annealing and polymerization at 60 °C and plate read for 5 s at 76 °C, followed by melt cycle. The PCR reaction mixture (15 μL) contained 1 μL cDNA (100 ng), 0.4 μL respective specific primers (10 pmol/μL), 6.1 μL water and 7.5 μL GoTaq^®^ qPCR Master Mix (Promega Corporation). qPCR reaction was performed on the AriaMx Real-time PCR System (Agilent, Santa Clara, CA, USA) and analyzed by Agilent Aria software version 1.5. Relative gene expression change was calculated according to the 2^−ΔΔCt^ method, where HPRT1 gene expression was taken as endogenous reference. Three independent experiments were performed and data expressed as means ± SEM. The significance of fold changes in gene expression between groups was analyzed using software tool REST (REST 2009-RG Mode, Qiagen) for group-wise comparison and statistical analysis of relative expression results in real-time PCR [88].

Student’s *t*-test or Mann-Whitney test (according to the results of Shapiro-Wilk normality test) applied to the ΔCt values. The primer sequences used for expression analysis: *CTNNB1* for: GCTATTGTAGAAGCTGGTGGAATGC, *CTNNB1* rev: CTTCCATCCCTT CCTGTTTAGTTGC (133 bp); *CCND1* for: TGAACTACCTGGACCGCTTC, *CCND1* rev: CCACTTGAGCTTGTTCACCA (206 bp); *HPRT1* for: GGACTAATTATGGACAGGACT, *HPRT1* rev: GCTCTTCAGTCTGATAAAATCTAC (195 bp).

### 4.9. Western Blot

Cultured cells (untreated or treated with PRI-724 for 3 days (NTERA-2 pair: 1.25 μM; NCCIT and JEG-3 pairs: 0.63 μM; TCam-2 pair: 2.5 μM)) were collected by trypsinization and cell pellets were resuspended in RIPA buffer (Cell Signaling Technology^®^, Danvers, MA, USA) containing Roche cOmplete™ Protease Inhibitor Cocktail (Sigma-Aldrich). Lysates were then centrifuged for 10 min at 14,000× *g* at 4 °C. Concentration of protein in supernatants was determined using Modified Lowry Protein Assay Kit (Thermo Scientific). Electrophoresis on gradient SDS polyacrylamide gels was used for the separation of protein extract from each sample and proteins were then transferred to Hybond PVDF blotting membrane (GE Healthcare, Life Sciences, Chicago, IL, USA) using semidry blotting (Owl,Inc., London, UK). One membrane was blocked in 5% non-fat dry milk in TBS-T for 1 h at room temperature and then incubated with primary β-catenin (#9562, Cell Signaling Technology; dilution 1:1000; 92 kDa) and cyclin D1 (#2926, Cell Signaling Technology; dilution 1:250; 36 kDa) antibodies in 5% BSA in TBS-T overnight at 4 °C. The second membrane was blocked in 5% non-fat dry milk in TBS-T overnight at 4 °C and then incubated with β-actin primary antibody (ab6276, Abcam, Cambridge, UK; dilution 1:5000; 42 kDa) for 1 h at room temperature. Horseradish peroxidase-linked secondary goat anti-mouse antibody (ab6789, Abcam) and chemiluminescence detection system (Luminata™ Crescendo Western HRP Substrate, Millipore, Burlington, MA, USA) were used for the visualization. Each membrane was digitally captured with C-DiGit imaging system (LI-COR, Lincoln, NE, USA) and β-catenin, cyclin D1 and β-actin densities were measured using Image Studio Digits Ver 5.2. software (Image Studio™ Lite Software, LI-COR).

### 4.10. In Vivo Experiments

6 to 8-week-old SCID beige mice (CD17 Cg-Prkdscid Lystbg/Crl, Charles River, Germany) or NSG mice (The Jackson Laboratory, Bar Harbor, ME, USA) were used in accordance with institutional guidelines under approved protocols. Project was approved by the Institutional Ethic Committee and by the national competence authority (State Veterinary and Food Administration of the Slovak Republic), registration No. Ro 1030/18-221 in compliance with the Directive 2010/63/EU and the Regulation 377/2012 on the protection of animals used for scientific purposes. It was performed in the approved animal facility (license No. SK UCH 02017).

To produce GCT cell lines xenografts for β-catenin immunohistochemical analysis, suspension of 2 × 10^6^ GCT cells, both parental and resistant, in 100 µL of extracellular matrix (ECM) mixture 1:1 (50 µL serum free DMEM medium, 50 µL ECM) was injected s.c. into the flank of NSG mouse. Xenografts were measured by caliper and animals were sacrificed at the point when the tumors exceeded 1 cm in diameter.

To test the effect of PRI-724 in vivo, suspension of 2 × 10^5^ NTERA-2 CisR in 100 µL of extracellular matrix (ECM) mixture 1:1 (50 µL serum free DMEM medium, 50 µL ECM) was injected s.c. into the flanks, in total 2 injections per SCID mouse. Mice were divided into four groups according to the treatment: cisplatin i.p./PRI-724 i.p./PRI-724 and cisplatin i.p./untreated controls. Tumors were measured by caliper and volume was calculated according to the formula for the volume of ellipsoid: volume = 0.52 × ((width + lenght)/2)^3^. Animals were sacrificed at the point when the tumors exceeded 1 cm in diameter. The results were evaluated as the mean of tumor volume.

### 4.11. Immunohistochemistry

Slides were deparaffinised, rehydrated and immersed in phosphate buffered saline solution (10 mM, pH 7.2). Tissue epitopes were demasked through revitalisation in TRIS-EDTA retrieval solution (10 mM TRIS, 1 mM EDTA, pH 9,0) at 98 °C for 20 min in Dako PT Link (Dako, Glostrup, Denmark). The slides were subsequently incubated for 1 h at room temperature with primary mouse monoclonal antibody against β-catenin (IR702, Ready-to-Use, Dako) and immunostained using anti-mouse/anti-rabbit secondary antibody (EnVision FLEX/HRP, Dako) for 30 min at room temperature. The reaction was visualised by diaminobenzidine substrate-chromogen solution (DAB, Dako) which was applied for 5 min. Ultimately, the slides were counterstained with hematoxylin. Non-neoplastic testicular tissue was used as a positive control and the same tissue without incubation in primary antibody represented the negative control. Representative images were captured with Olympus BX40 microscope (Olympus Corporation, Tokyo, Japan) and Canon EOS 1000D (Canon Inc., Tokyo, Japan).

### 4.12. Statistical Analysis

For the statistical analysis of studies involving comparison between the two groups, the normality assumption hypothesis was tested using Shapiro-Wilk test and differences were assessed by Student’s *t*-test or Mann-Whitney U test depending on normality of the data. The effect of tested drugs in vivo and the results of wound healing assay were analyzed using multivariate analysis. GraphPad Prism^®^ software (GraphPad Inc., La Jolla, CA, USA) was used. The *p*-values with *p* < 0.05 were considered to be statistically significant.

## 5. Conclusions

In summary, we herein characterize the effects of the Wnt signaling inhibitors—PRI-724 and LGK974 on parental and cisplatin-resistant GCT cell lines. LGK974 was not more cytotoxic for resistant cells compared to sensitive ones. The inhibitor of CBP/β-catenin complex PRI-724 seemed to be promising only in the treatment of cisplatin-resistant NTERA-2 CisR cells, where it negatively affected cell viability, probably through strong activation of caspase-3/7. PRI-724 also decreased the migratory capacity and tumorigenicity of NTERA-2 CisR cell line. PRI-724 inhibitor was not effective in cisplatin-resistant GCT cell lines with increased expression of β-catenin and cyclin D1 suggesting involvement of these two proteins in resistance to PRI-724 inhibition. We showed that Wnt/β-catenin signaling is deregulated in cisplatin-resistant GCT cell lines. However, PRI-724 and LGK974 inhibitors did not seem to be effective in the treatment of chemoresistant GCT cell lines, except for NTERA-2 CisR cells. Nevertheless, our data indicated that inhibition of this pathway could be beneficial in the treatment of refractory GCT patients, and further research is therefore warranted.

## Data Availability

Data is contained within the article or supplementary material.

## Supplementary Materials

The following are available online at https://www.mdpi.com/article/10.3390/ijms22084263/s1.
