# Supplementary material for: Targeting of Deregulated Wnt/β-Catenin Signaling by PRI-724 and LGK974 Inhibitors in Germ Cell Tumor Cell Lines
Source: Int J Mol Sci. 2021 Apr 20;22(8):4263. doi: 10.3390/ijms22084263 (PMC8073733; doi:10.3390/ijms22084263)
Supplement: Supplementary file 1 [file ijms-22-04263-s001.zip › Supplementary Files/Supplementary Figures/Supplementary Figures.docx]

Supplementary materials

**Targeting of deregulated Wnt/β-catenin signaling by PRI-724 and LGK974 inhibitors in germ cell tumor cell lines**

**
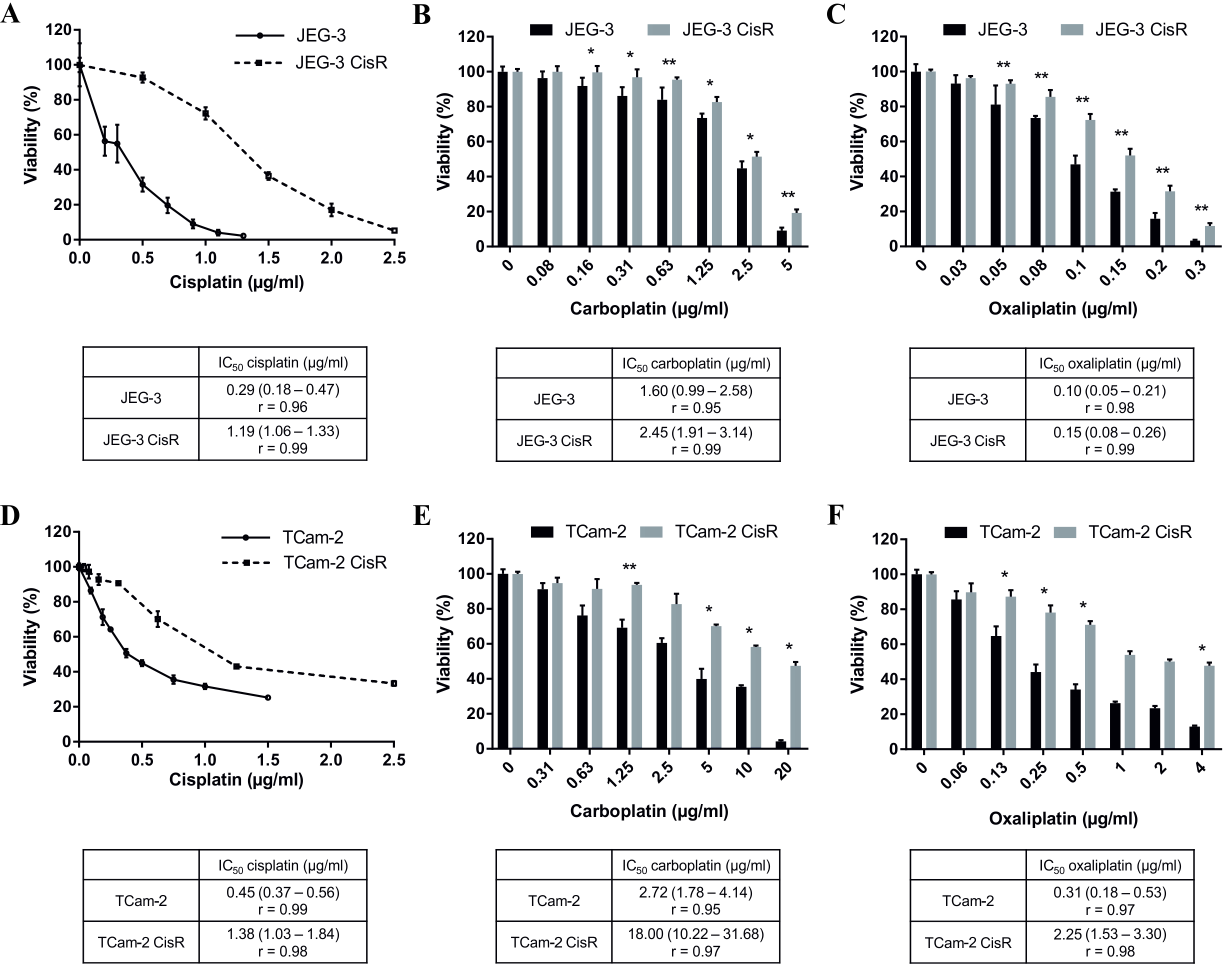
**

**Supplementary Figure S1.** Cisplatin-resistant JEG-3 CisR and TCam-2 CisR cell lines exhibited cross-resistance to platinum drugs. (A–C) Cytotoxicity of cisplatin, carboplatin and oxaliplatin in JEG-3 CisR cells was substantially decreased in comparison to parental cell line. (D-E) Resistance to cisplatin, carboplatin and oxaliplatin was significantly increased in TCam-2 CisR cells. Relative viability was determined by luminescent viability assay on day 6 (carboplatin, oxaliplatin) or 7 (cisplatin). Values were expressed as the averages of quadruplicates ± SD and IC_50_ values were stated in tables below graphs. * p < 0.05, ** p < 0.01


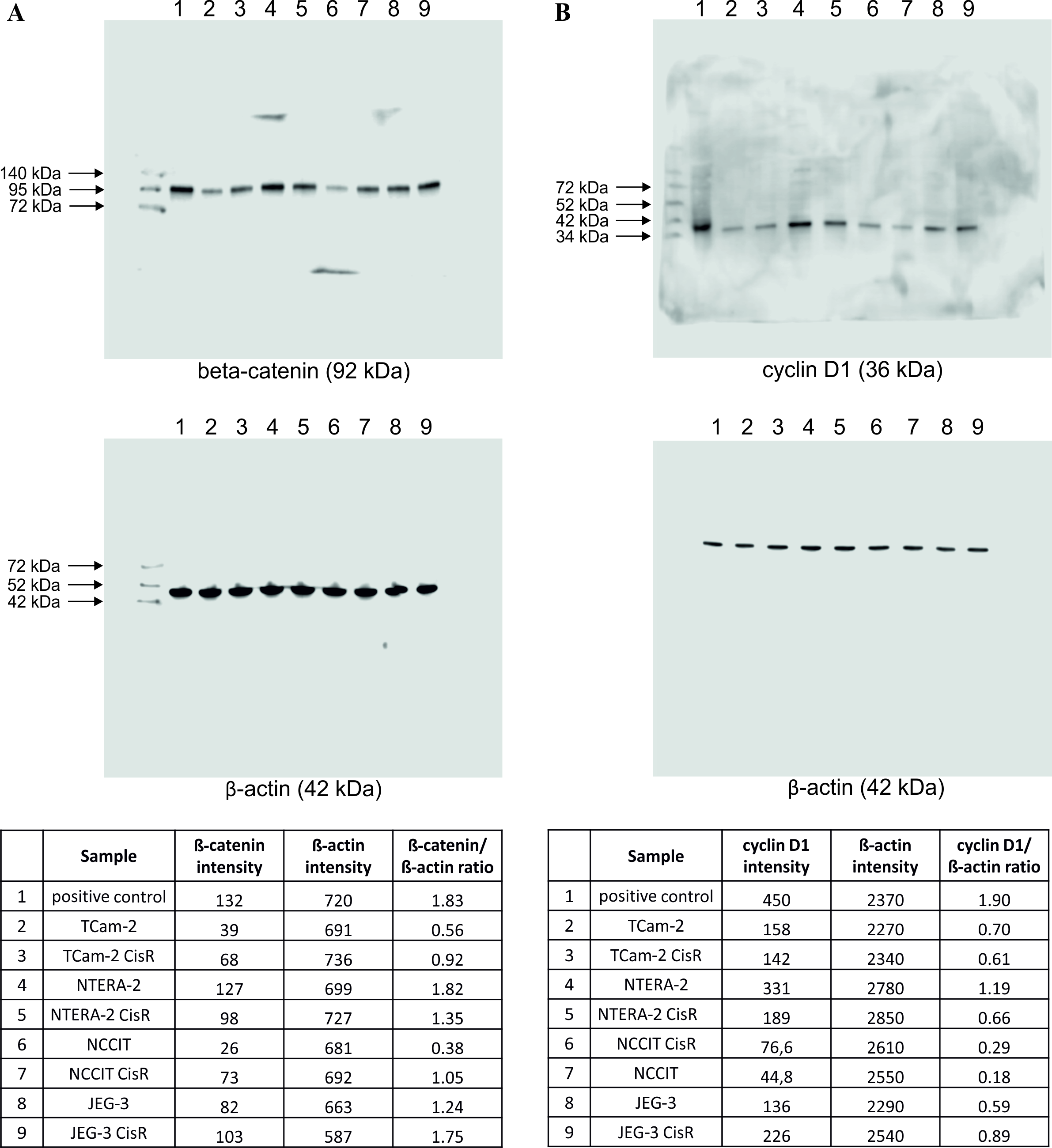


**Supplementary Figure S2.** Western blot analysis of β-catenin, cyclin D1 and β-actin levels in different GCT cell lines. (A) Western blot analysis of β-catenin showed increased level of this protein in TCam-2 CisR and NCCIT CisR cells. Decreased β-catenin level was present in NTERA-2 CisR cell line compared to parental cells. (B) NTERA-2 CisR cells exhibited also decreased level of cyclin D1.

β-catenin, cyclin D1 and β-actin intensities, and their ratios for each cell line are listed in the tables.


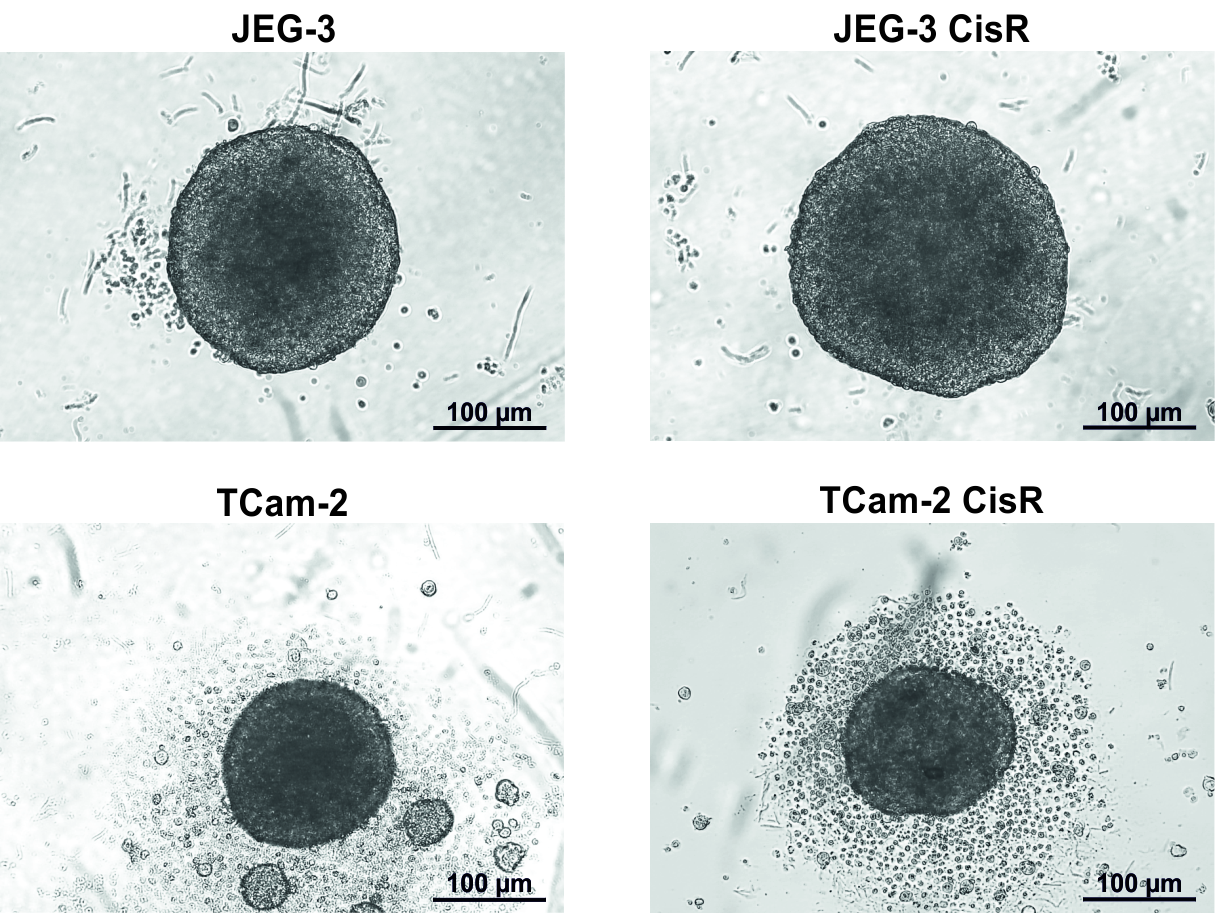


**Supplementary Figure S3.** GCT cell lines JEG-3, TCam-2 and their cisplatin-resistant variants were able to form 3D multicellular spheroids, when seeded into ultra-low attachment round bottom plates.


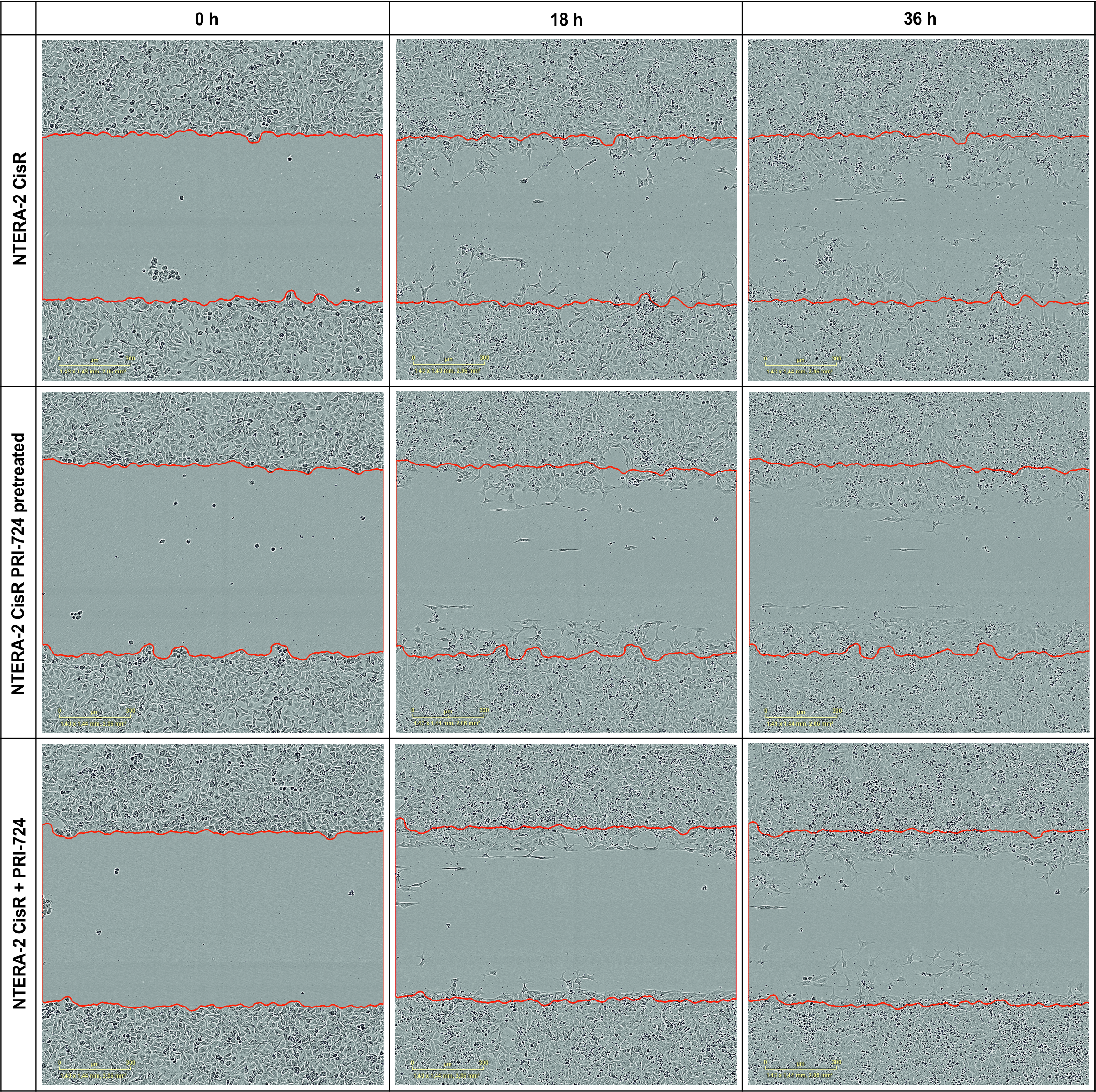


**Supplementary Figure S4.** Migration of NTERA-2 CisR cells was decreased when cells were pretreated (for 72 h) or treated (for 36 h after the wound was made) with PRI-724 inhibitor. Confluent monolayers of cells were wounded and cell migration was observed by live-cell imaging for 36 h. Representative pictures were shown at time 0 h, 18 h and 36 h. Red line – initial scratch wound line.


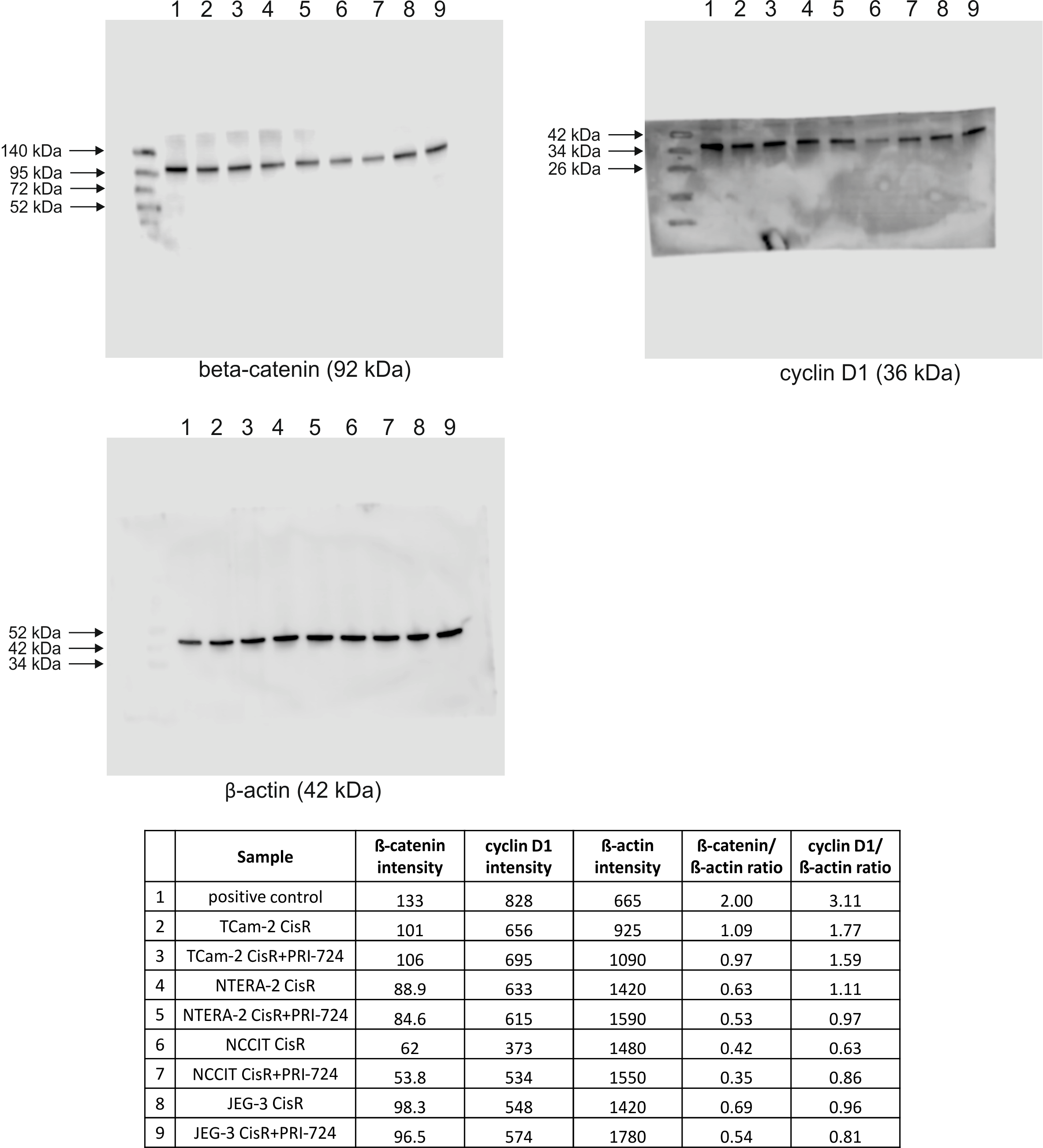


**Supplementary Figure S5.** Western blot analysis of β-catenin, cyclin D1 and β-actin levels revealed no significant changes in cisplatin-resistant GCT cell lines treated with PRI-724. β-catenin, cyclin D1 and β-actin intensities, and their ratios for each cell line with and without PRI-724 treatment are listed in the table.
