# Supplementary figures and images for: Targeting of Deregulated Wnt/β-Catenin Signaling by PRI-724 and LGK974 Inhibitors in Germ Cell Tumor Cell Lines
Source: Int J Mol Sci. 2021 Apr 20;22(8):4263. doi: 10.3390/ijms22084263 (PMC8073733; doi:10.3390/ijms22084263)

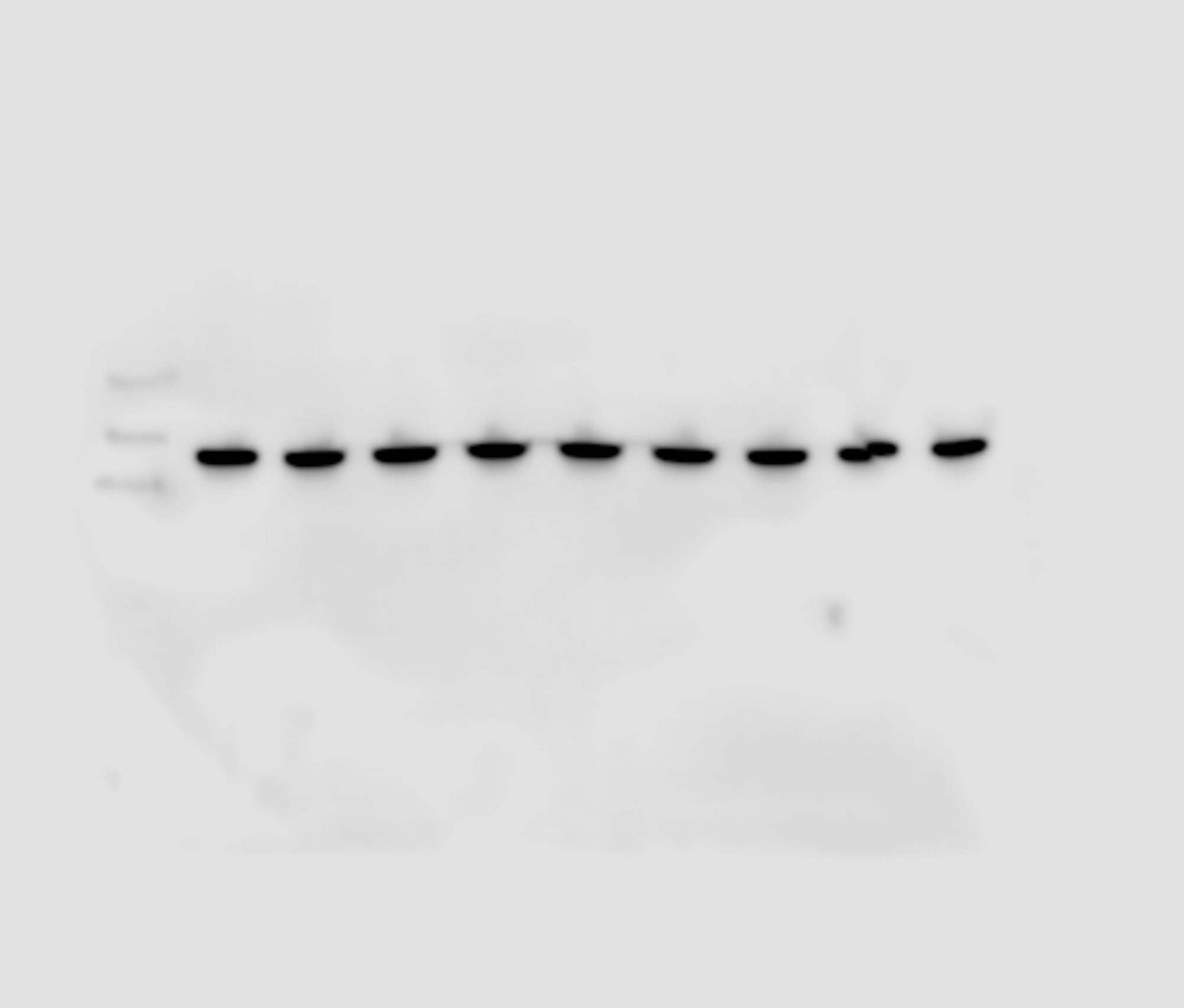

Supplement: Supplementary file 1 [file ijms-22-04263-s001.zip › Supplementary Files/Western Blots/WB1 beta aktin - parental vs resistant.tif]

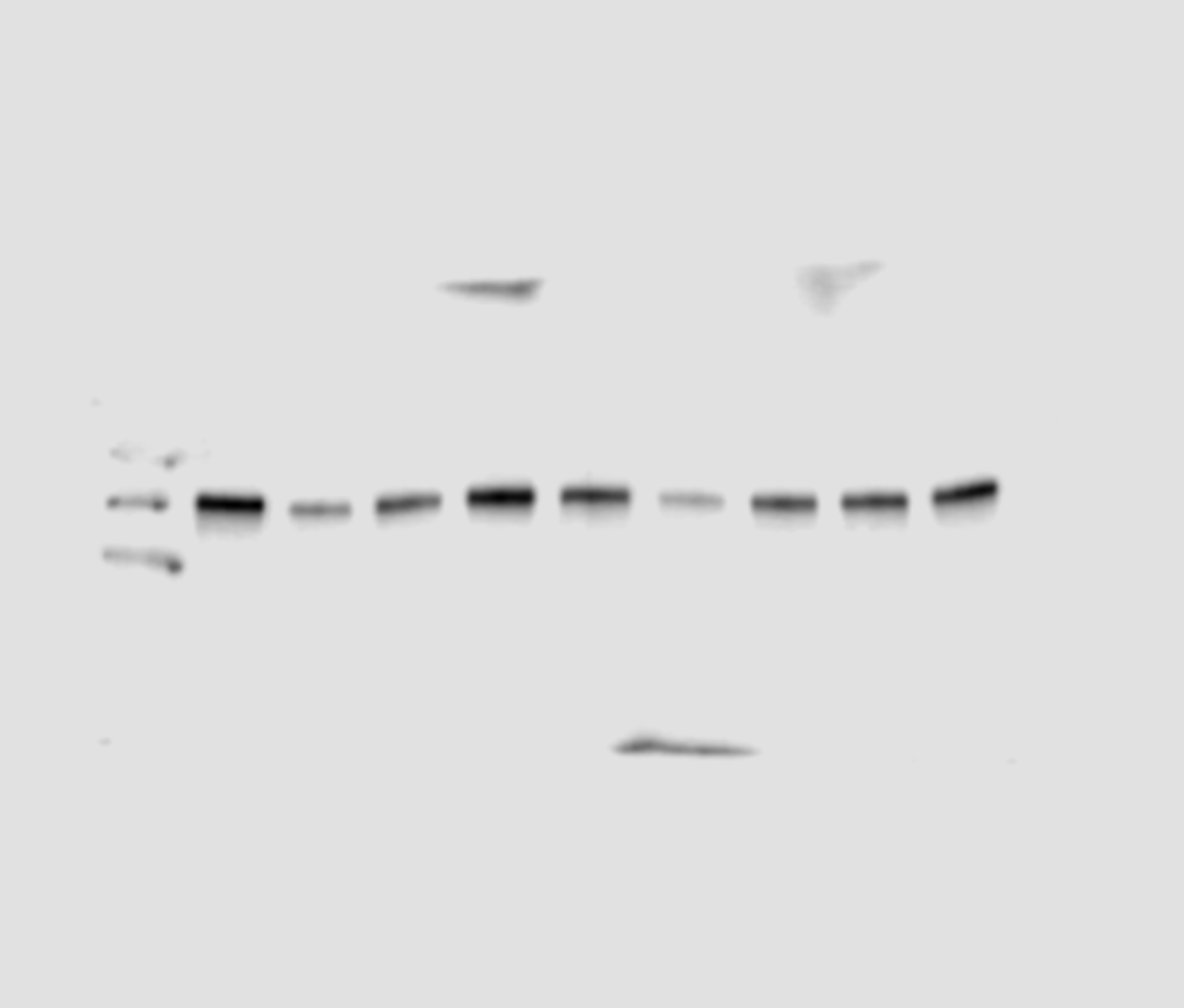

Supplement: Supplementary file 1 [file ijms-22-04263-s001.zip › Supplementary Files/Western Blots/WB1 beta catenin - parental vs resistant.tif]

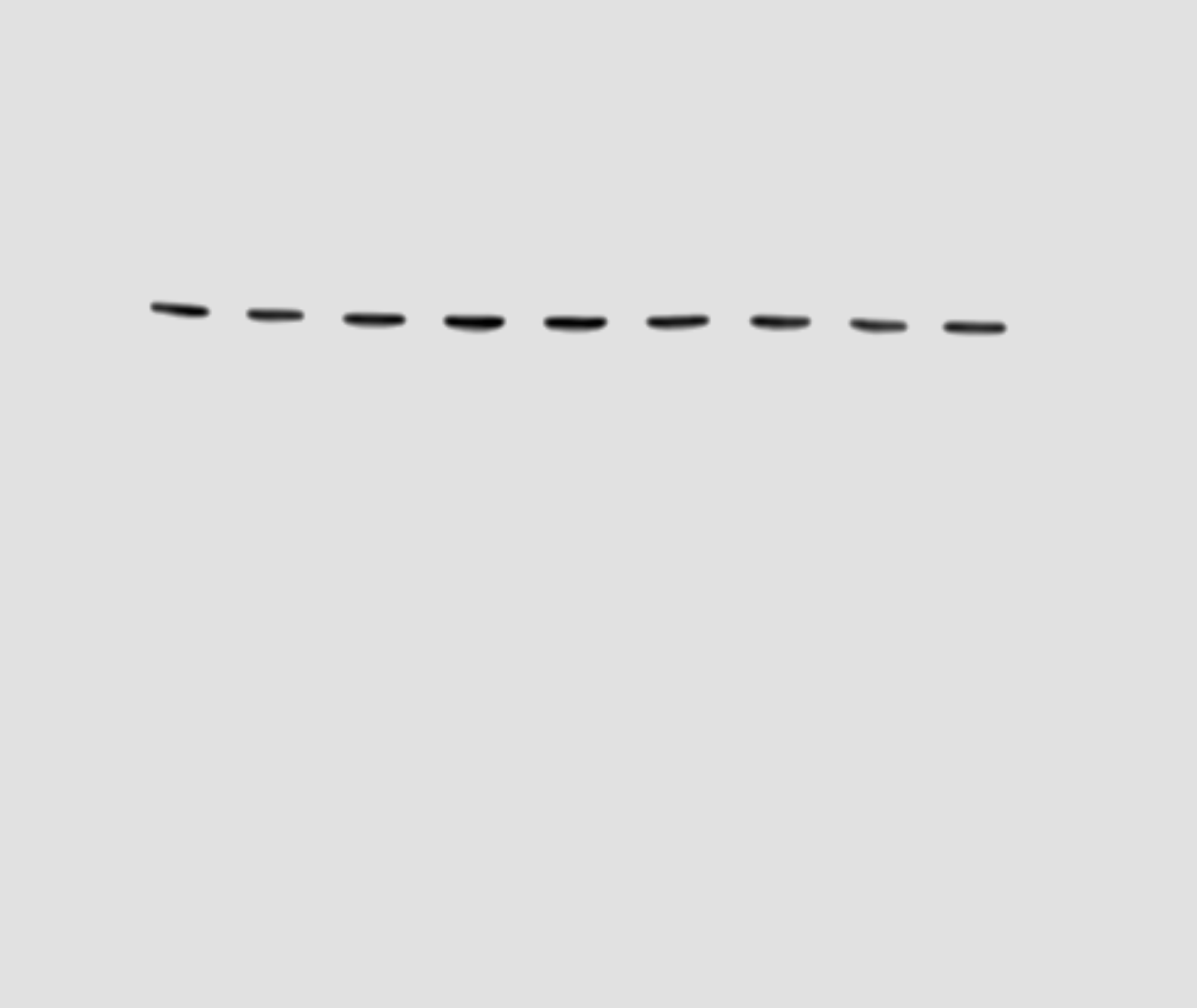

Supplement: Supplementary file 1 [file ijms-22-04263-s001.zip › Supplementary Files/Western Blots/WB2 beta actin - parental vs resistant.tif]

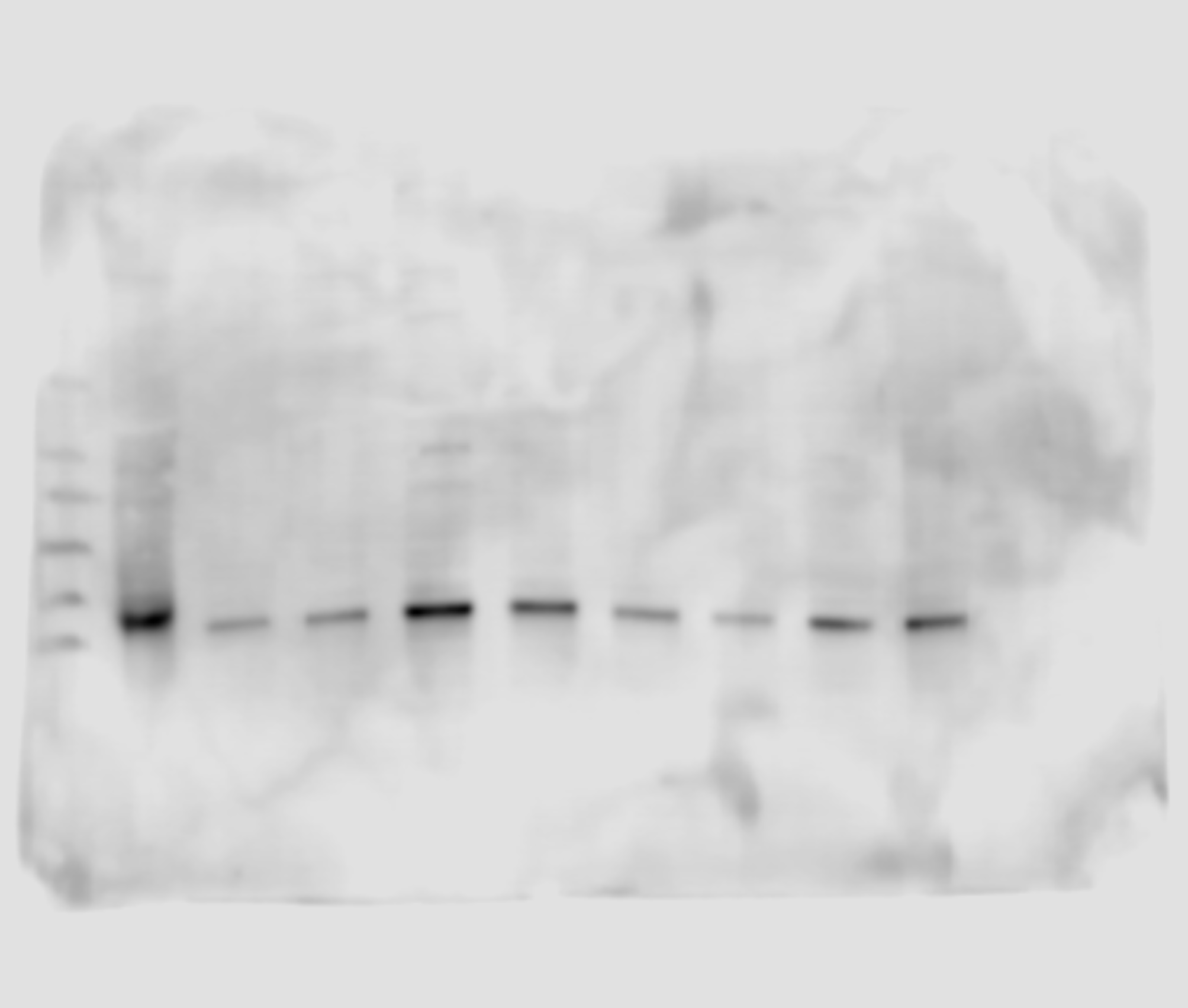

Supplement: Supplementary file 1 [file ijms-22-04263-s001.zip › Supplementary Files/Western Blots/WB2 cyclin D1 - parental vs resistant.tif]

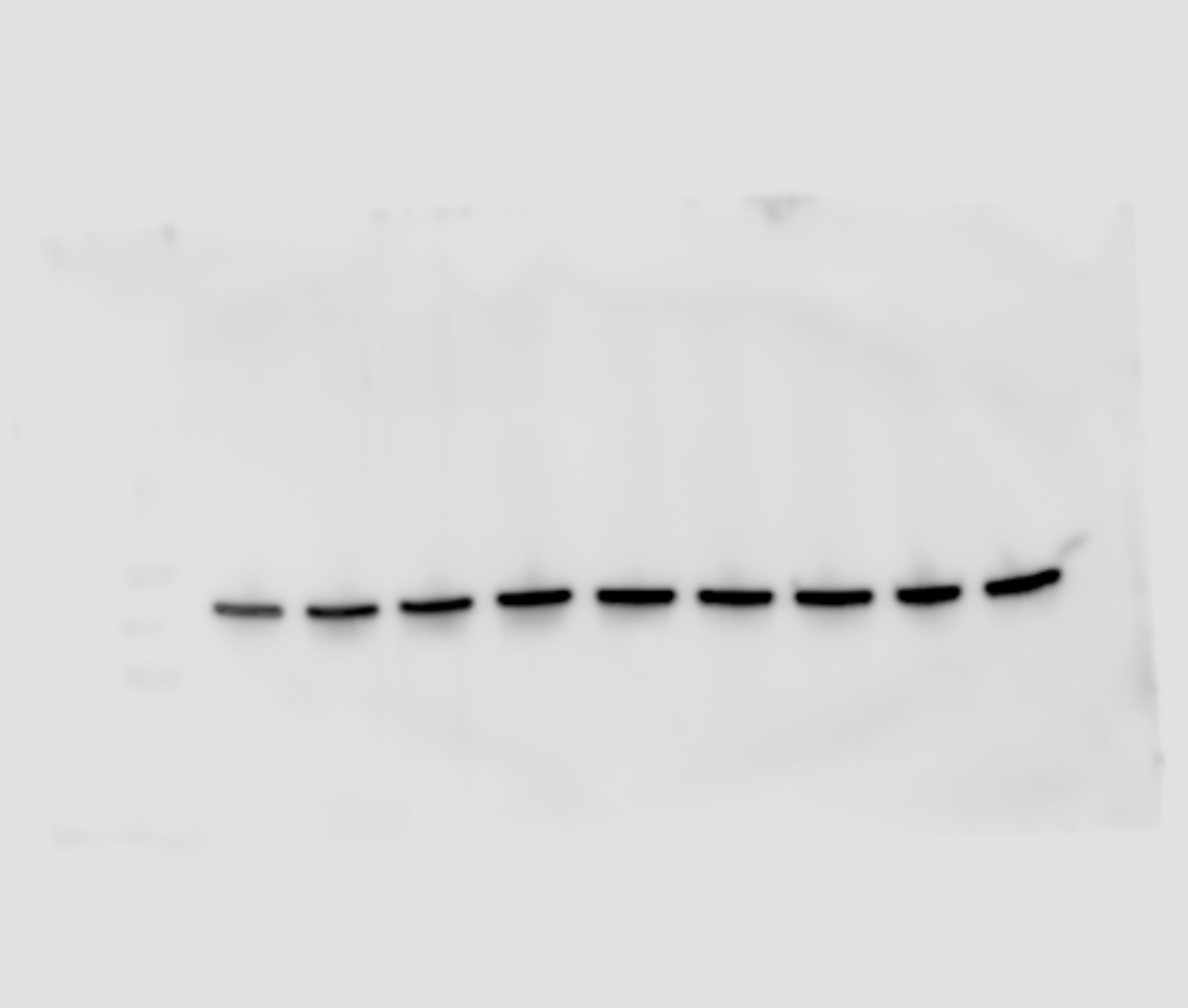

Supplement: Supplementary file 1 [file ijms-22-04263-s001.zip › Supplementary Files/Western Blots/WB3 beta actin - resistant vs treated.tif]

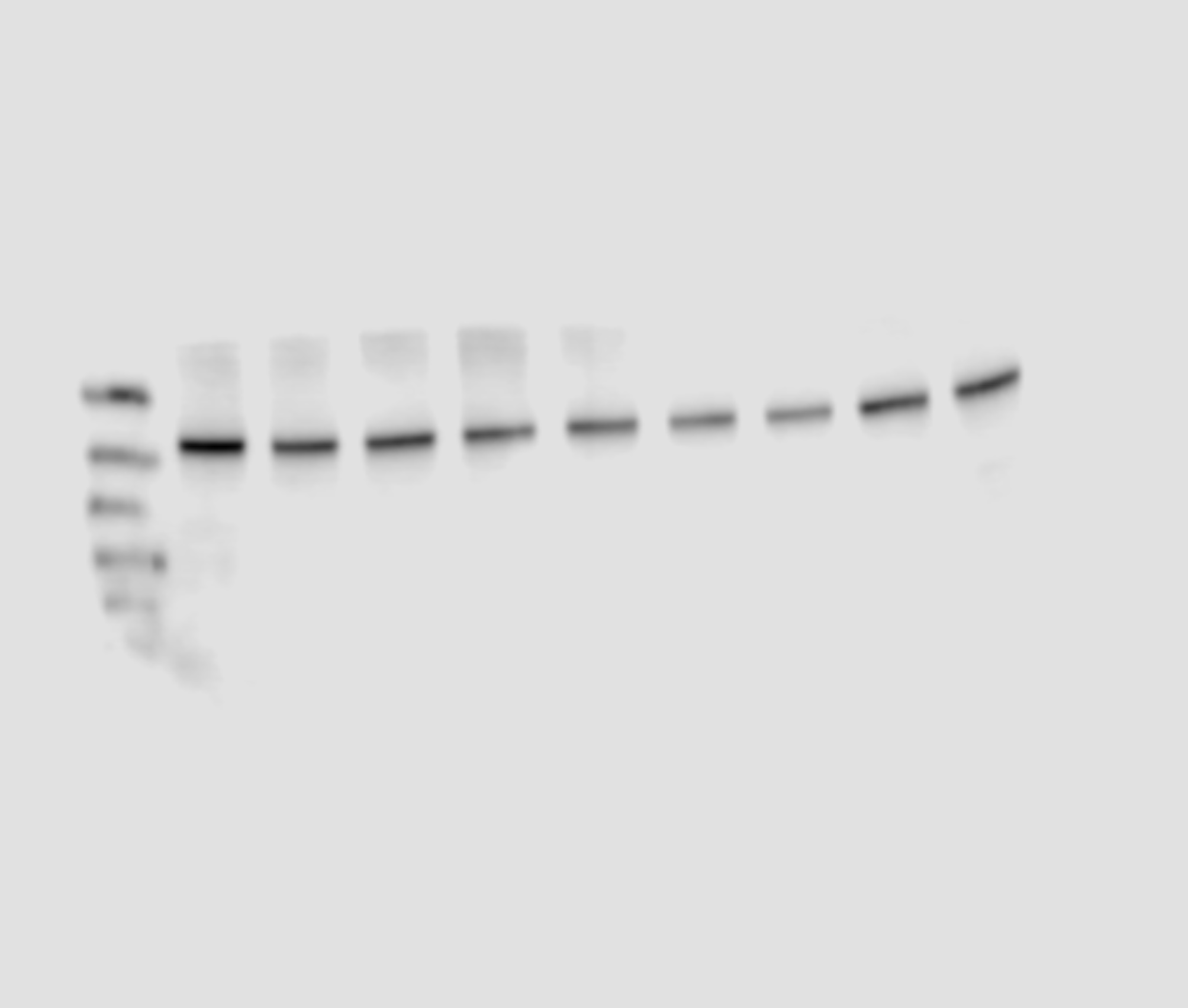

Supplement: Supplementary file 1 [file ijms-22-04263-s001.zip › Supplementary Files/Western Blots/WB3 beta catenin - resistant vs treated.tif]

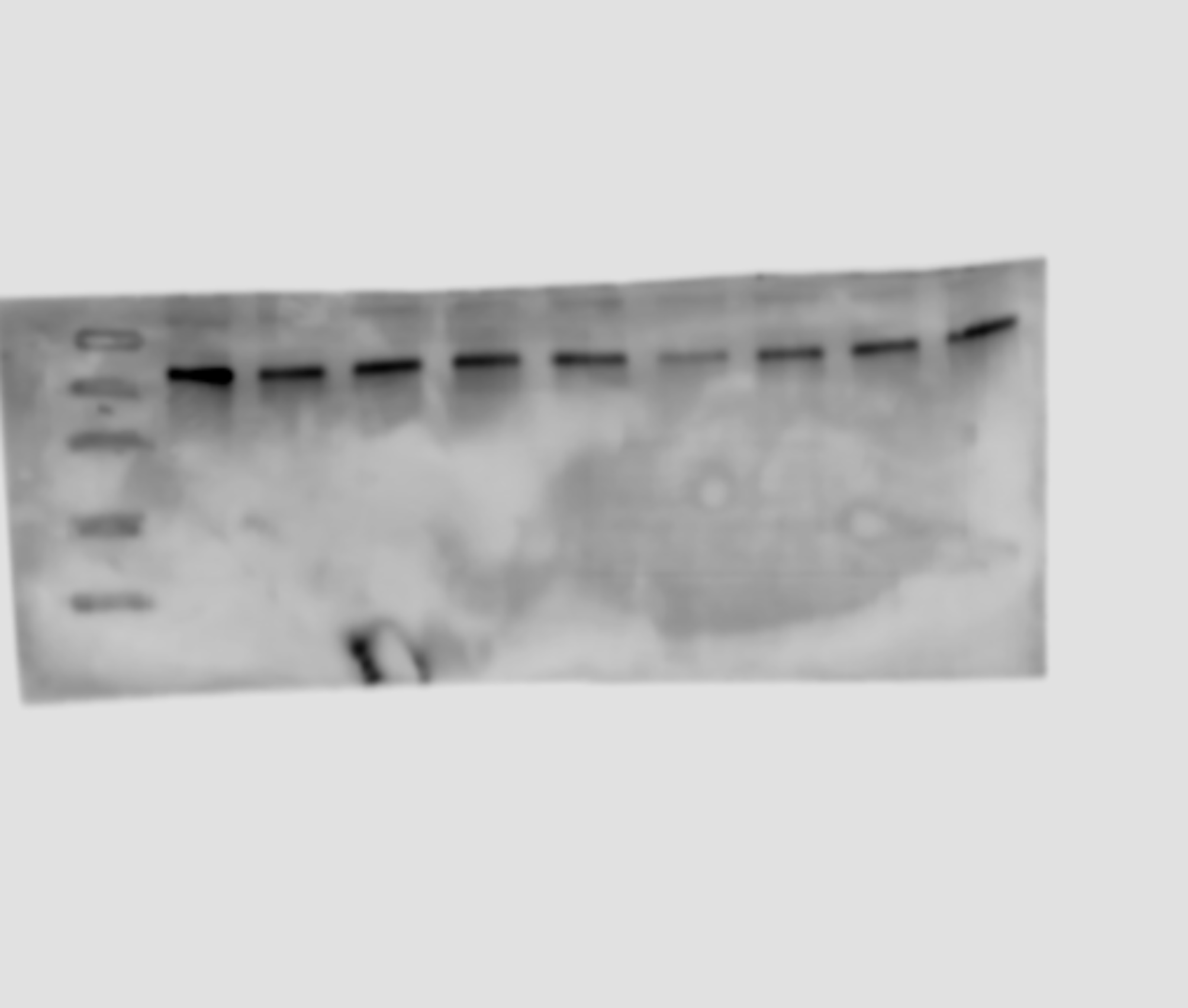

Supplement: Supplementary file 1 [file ijms-22-04263-s001.zip › Supplementary Files/Western Blots/WB3 cyclin D1 - resistant vs treated.tif]
